# Expression of AXL receptor tyrosine kinase relates to monocyte dysfunction and severity of cirrhosis

**DOI:** 10.26508/lsa.201900465

**Published:** 2019-12-10

**Authors:** Robert Brenig, Oltin T Pop, Evangelos Triantafyllou, Anne Geng, Arjuna Singanayagam, Christian Perez-Shibayama, Lenka Besse, Jovana Cupovic, Patrizia Künzler, Tuyana Boldanova, Stephan Brand, David Semela, François HT Duong, Christopher J Weston, Burkhard Ludewig, Markus H Heim, Julia Wendon, Charalambos G Antoniades, Christine Bernsmeier

**Affiliations:** 1 Department of Biomedicine, University of Basel and University Centre for Gastrointestinal and Liver Diseases, Basel, Switzerland; 2 Medical Research Centre and Division of Gastroenterology and Hepatology, Cantonal Hospital St. Gallen, St. Gallen, Switzerland; 3 Institute of Liver Studies, King’s College Hospital, King’s College London, London, UK; 4 Hepatology Department, St. Mary’s Hospital, Imperial College London, London, UK; 5 Institute of Immunobiology, Medical Research Centre, Cantonal Hospital St. Gallen, St. Gallen, Switzerland; 6 Laboratory of Experimental Oncology, Department of Oncology and Haematology, Cantonal Hospital St. Gallen, St. Gallen, Switzerland; 7 Centre for Liver Research and National Institute for Health Research, Biomedical Research Unit, University of Birmingham, Birmingham, UK

## Abstract

Infectious complications in patients with cirrhosis frequently initiate episodes of decompensation and substantially contribute to the high mortality. Mechanisms of the underlying immuneparesis remain underexplored. TAM receptors (TYRO3/AXL/MERTK) are important inhibitors of innate immune responses. To understand the pathophysiology of immuneparesis in cirrhosis, we detailed TAM receptor expression in relation to monocyte function and disease severity prior to the onset of acute decompensation. TNF-α/IL-6 responses to lipopolysaccharide were attenuated in monocytes from patients with cirrhosis (n = 96) compared with controls (n = 27) and decreased in parallel with disease severity. Concurrently, an AXL-expressing (AXL^+^) monocyte population expanded. AXL^+^ cells (CD14^+^CD16^high^HLA-DR^high^) were characterised by attenuated TNF-α/IL-6 responses and T cell activation but enhanced efferocytosis and preserved phagocytosis of *Escherichia coli*. Their expansion correlated with disease severity, complications, infection, and 1-yr mortality. AXL^+^ monocytes were generated in response to microbial products and efferocytosis in vitro. AXL kinase inhibition and down-regulation reversed attenuated monocyte inflammatory responses in cirrhosis ex vivo. AXL may thus serve as prognostic marker and deserves evaluation as immunotherapeutic target in cirrhosis.

## Abstract (precis)

Immune dysfunction determines morbidity and mortality in liver cirrhosis. Distinct AXL-expressing circulating monocytes, which regulate antimicrobial responses, expand with progression of the disease.

## Graphical abstract

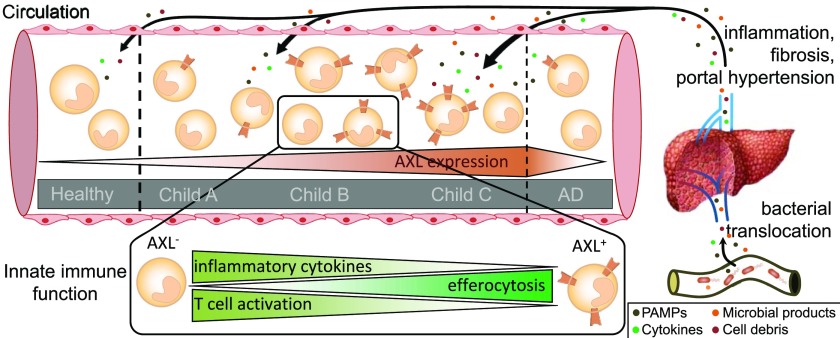

## Introduction

Patients with cirrhosis are at increased risk of infection and consequent acute decompensation (AD) with substantially elevated morbidity and mortality (1). Compared with the overall rate of infections in hospitalised patients (5–7%), bacterial infections occur significantly more frequently in patients with cirrhosis (32–34%) (2, 3). Similarly, infections account for more than 50% of hospitalisations in cirrhotic patients, are the main precipitant for AD without and with organ failure (acute-on-chronic liver failure [ACLF]) (4, 5), and implicate a high mortality (2, 6). Infection susceptibility in cirrhosis has been attributed to a state of immuneparesis, defined by inadequate immune responses to microbial challenge (7, 8, 9).

The pathophysiology of immuneparesis in cirrhosis is highly complex and remains incompletely understood, involving diverse defects in immune cell function, including monocytes, and soluble factors in multiple compartments (7). Circulating monocytes from patients with AD and ACLF compared with stable cirrhosis demonstrated reduced expression of HLA-DR and attenuated production of TNF-α/IL-6 in response to lipopolysaccharide (LPS), which has previously been linked to adverse outcome (10, 11, 12). Moreover, the role of bacterial translocation in the pathogenesis of immune dysfunction and infection susceptibility has been highlighted (13, 14).

TAM receptors (TYRO3, AXL, and MERTK) belong to the family of receptor tyrosine kinases. Among immune cells, they are expressed on monocytes, macrophages, dendritic cells, and glial cells, and additionally on epithelial cells of the reproductive system, the retina, and tumour cells (15). TAM receptors are important regulators of innate immune homeostasis, acting by inhibition of TLR signalling pathways through a signal transducer and activator of transcription 1 (STAT1)- and suppressors of cytokine signalling (SOCS1/3)-dependent mechanism (15, 16) and by promotion of phagocytic removal of apoptotic cells (efferocytosis) (16). Their activation succeeds ligand binding (growth arrest–specific gene-6 [GAS6], PROTEIN S) and interaction with phosphatidylserine on apoptotic cells (15, 16, 17). In murine dendritic cells, activation required interaction with the type I interferon receptor (IFNAR) (16).

We recently identified the expansion of MERTK-expressing monocytes and macrophages in diverse compartments in patients with ACLF that dampened innate immune responses to microbial challenge and conferred disease severity and adverse outcomes (18). The expansion of MERTK-expressing monocytes and macrophages was moreover detected in acute liver failure (18) and characterised by both suppressed immune responses and enhanced efferocytic capacities (19). Another immune-suppressive population, expanded in the circulation of patients with ACLF, was monocytic myeloid–derived suppressor cells (M-MDSC) that suppressed T cell activation, innate immune responses, and pathogen uptake (20).

It is not clear when and/or under which circumstances immuneparesis and monocyte dysfunction occurs with an associated susceptibility to infection during the clinical course of cirrhosis and portal hypertension, before the onset of AD. The main emphasis of this study is to detail the expression of TAM receptors on monocytes in relation to monocyte function and disease severity of patients with cirrhosis in the absence of AD using patients with AD, chronic liver disease (CLD) without cirrhosis and healthy controls (HCs) as comparators. We hereby seek to better understand the pathophysiology of immuneparesis development in patients with cirrhosis prior to AD and identify candidates for biomarkers and future immunotherapeutic targets that may preserve innate immune responses.

## Results

### Patient characteristics

Patients with cirrhosis were distinguished between Child-Pugh A, B, and C and compared with AD, CLD without cirrhosis, and HC. The cohort was characterised by disease severity scores, aetiologies, and diverse clinical parameters (Tables S1 and S2). In patients with cirrhosis without AD, 1-yr mortality rate was 4.5% and rising with Child-Pugh stage: A (0%), B (5.7%), and C (11.8%). 1-yr mortality rate for those with AD was 75%, with two of eight patients dying within 28 d of enrolment. N = 9 patients deceased from cirrhosis-related complications during follow-up of 1 yr (secondary infections [n = 4], ACLF with multiorgan failure [n = 3], hepatocellular carcinoma [HCC] [n = 1], hypovolemic shock due to variceal bleeding [n = 1]), and the cause of one death was unknown. Current infections at hospital admission were seen in 62.5% of patients with AD. Within 4 wk following inclusion into the study, 5.2% of patients (Child B: 5.7%, Child C: 5.9%, and AD 25%) developed infectious complications, adapted from the definition by Bajaj et al (9). Episodes of AD developed in 10.2% of cirrhotic patients (Child B: 17.1% and Child C: 17.6%) within 4 mo following inclusion (Table S1).

Table S1 Clinical characteristics for different groups of patients and HCs at the time of blood sampling and experimental ex vivo assays.

Table S2 Disease severity scores and the underlying aetiology for different groups of patients at the time of blood sampling.

### Innate immune responses are impaired in patients with cirrhosis and parallel the expansion of an AXL-expressing circulating monocyte population

**Figure 1.**
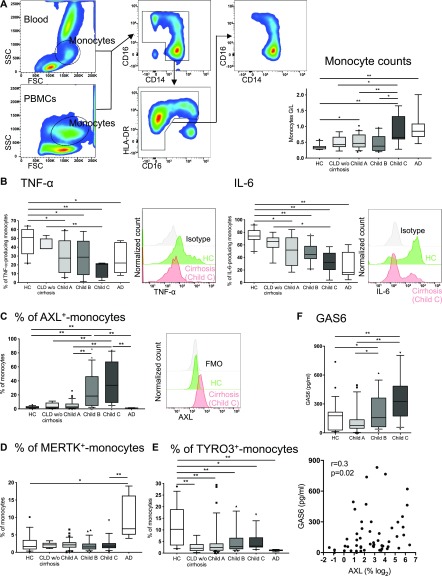
TAM (TYRO3, AXL, and MERTK) receptor expression and functional characterisation of circulating monocytes in cirrhosis. **(A)** FACS gating strategy used to identify circulating monocytes in whole blood or PBMCs (left panel). Side scatter (SSC), forward scatter (FSC). Monocyte counts (differential leucocyte count, right panel). **(B)** TNF-α– and IL-6–producing monocytes (%) in response to LPS ex vivo at different stages of cirrhosis and representative FACS histograms (HC, Child C, and isotype). **(C, D, E)** TAM receptor expression on circulating monocytes (%) at different stages of cirrhosis and representative FACS histograms for AXL expression (HC, Child C, and fluorescence minus one). **(F)** GAS6 levels (pg/ml) in HC and cirrhosis (upper panel) and in correlation with AXL expression (% of monocytes, lower panel). HC n = 27, CLD without (w/o) cirrhosis n = 8, Child A n = 36, Child B n = 28, Child C n = 17, and acute decompensation (AD) of cirrhosis n = 8. Data are presented as box plots showing median with 10–90 percentile. *P < 0.05/**P < 0.01 (Mann–Whitney tests, Spearman correlation coefficient).

In patients with AD/ACLF, we recently described impaired inflammatory cytokine production of circulating monocytes to microbial challenges (18, 20). Attenuated responses were also seen in stable cirrhotic patients (18, 20). It, however, remained unknown when and to what extent circulating monocytes develop immune dysfunction over the time course of disease progression. We measured ex vivo inflammatory cytokine production upon LPS treatment of circulating monocytes from patients with cirrhosis at different stages of disease. TNF-α and IL-6 production was reduced in cirrhosis compared with HC and incrementally decreased from Child A to C, and AD but remained preserved in patients with CLD without cirrhosis (Fig 1A and B).

**Figure S1.**
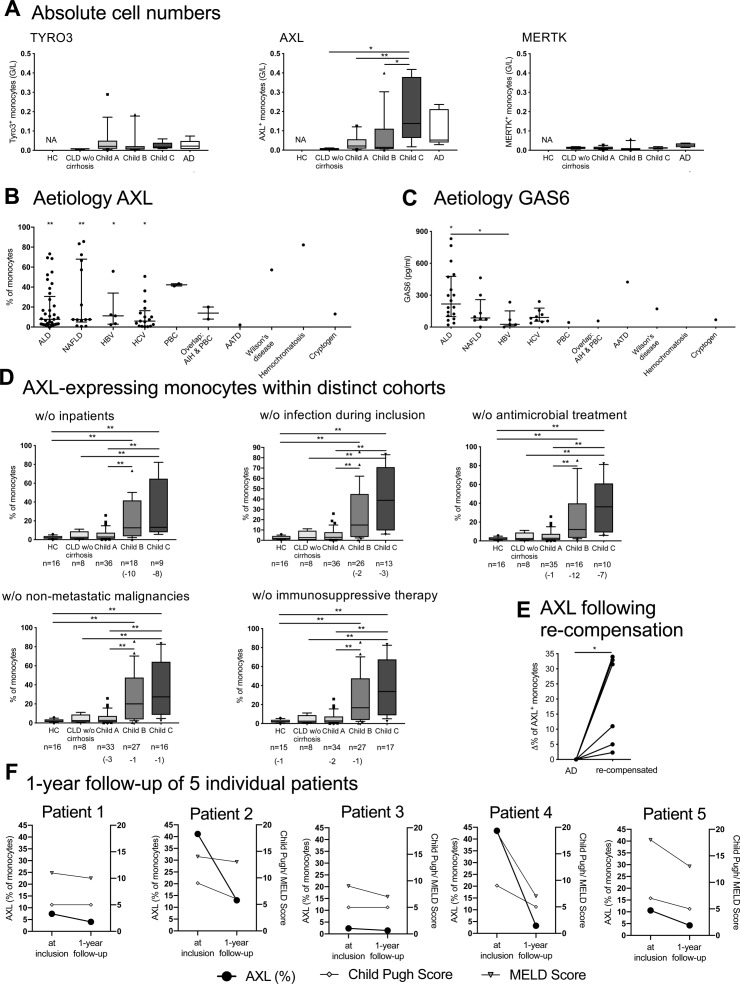
Numbers of TAM receptor-expressing monocytes in patients with cirrhosis, underlying aetiologies, cohorts of patients, and follow-up data of AXL-expressing monocytes. **(A)** Counts of TYRO3-, AXL-, and MERTK-expressing monocytes (G/L) in HCs and patients with cirrhosis (CLD without [w/o] cirrhosis, n = 5; Child A, n = 5; B, n = 11; C, n = 7; AD, n = 8). Median/10–90 percentile (Mann–Whitney tests). **(B, C)** Percentage of AXL-expressing monocytes and plasma ligand GAS6 levels (pg/ml) in different underlying aetiologies of cirrhosis. Alcoholic liver disease (AXL n = 37/ GAS6 n = 18); nonalcoholic fatty liver disease (n = 14/n = 8); hepatitis B virus (n = 7/n = 5); hepatitis C virus (n = 17/n = 10); primary biliary cholangitis (PBC; n = 2/n = 1); autoimmune hepatitis & PBC (AIH & PBC; n = 2/n = 1); alpha-1 antitrypsin deficiency (n = 1/n = 1); Wilson’s disease (n = 1/n = 1); hemochromatosis (n = 1/n = 1); and cryptogenic cirrhosis (n = 1/n = 1). Median with IQR. Statistical significance levels compared with HC and between aetiologies (Mann–Whitney tests). **(D)** AXL-expressing monocytes after the exclusion of distinct cohorts of patients. Median/10–90 percentile (Mann–Whitney tests). **(E, F)** Follow-up assessment of AXL-expressing monocytes of individual patients (E; re-compensation after AD [n = 6; n = 2 died during AD], F; 1 yr after inclusion showing Child-Pugh and MELD scores in parallel). *P < 0.05, **P < 0.01 (Wilcoxon test).

**Figure S2.**
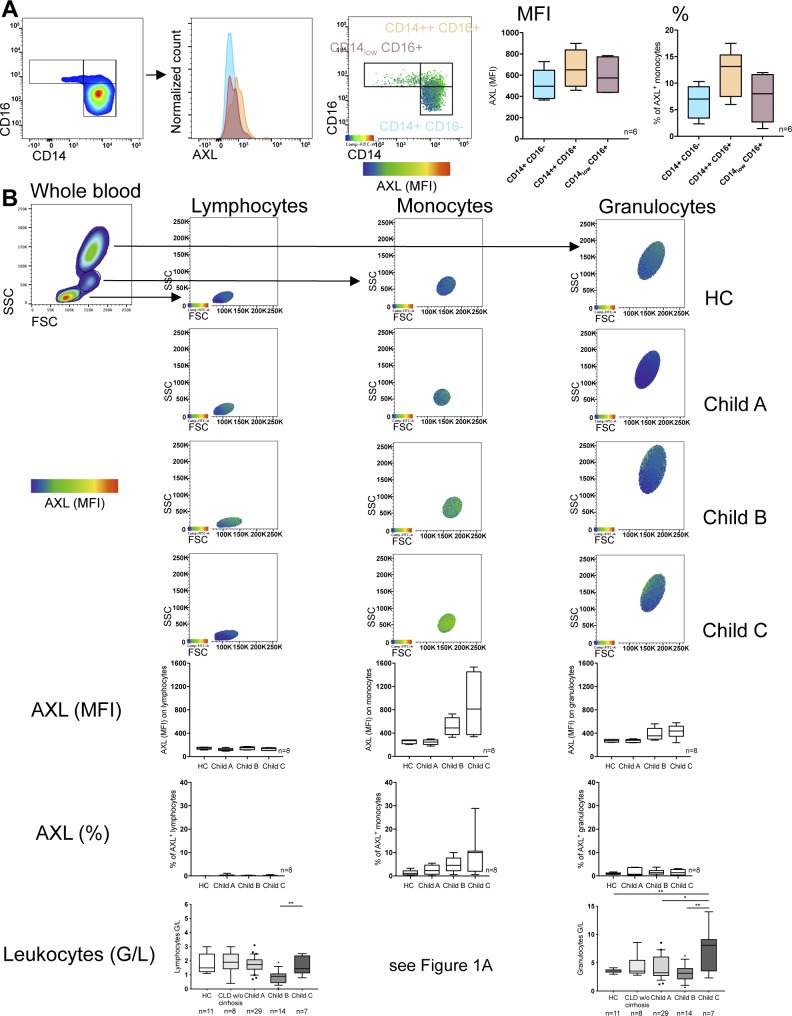
AXL expression levels on circulatory monocyte subsets and other leukocytes. **(A)** AXL expression on monocytes illustrated by a representative flow cytometry histogram, flow cytometry viSNE (visualization tool for high-dimensional single-cell data based on the t-Distributed Stochastic Neighbor Embedding [t-SNE] algorithm) (50), analysis of cirrhotic monocytes illustrating AXL expression on classical (CD14^+^CD16^−^), intermediate (CD14^++^CD16^+^), and nonclassical (CD14_low_CD16^+^) subsets, and its corresponding quantification shown in percentage and MFI. **(B)** Representative flow cytometry viSNE analyses and quantification (% of monocytes and MFI) for HCs, patients with CLD without (w/o) cirrhosis, and patients with cirrhosis Child A, B, and C showing AXL expression on different leukocytes such as monocytes, lymphocytes, and granulocytes. Leukocyte count (G/L). Side scatter (SSC); forward scatter (FSC). Median/10–90 percentile. *P < 0.05, **P < 0.01 (Mann–Whitney test).

In parallel with increased disease severity and the decline of inflammatory cytokine production in response to LPS, we demonstrated the expansion of an AXL-expressing monocyte population ex vivo in the circulation of patients with cirrhosis (Figs 1C and S1A). The occurrence of AXL-expressing monocytes was independent of the underlying aetiology and other potential confounders (inpatient treatment, current infection, antimicrobial treatment, immunosuppressive therapy, and non-metastatic malignancies; Fig S1B and D). Within monocyte subsets, the expression of AXL was highest in but not restricted to the intermediate subset (cluster of differentiation [CD]14^++^CD16^+^) (Fig S2A). AXL expression on monocytes of patients with CLD without cirrhosis was low; a similar pattern was also seen in AD (Fig 1C). Other immune cells such as lymphocytes and granulocytes barely expressed AXL (Fig S2B). Longitudinal follow-up data showed an increase in AXL expression after re-compensation of AD episodes and a change in AXL expression paralleling the evolution of disease severity after 1 yr (Fig S1E and F). Recently, we described a MERTK-expressing monocyte population that was expanded in the circulation of patients with AD/ACLF (18), which was again confirmed in this cohort (Fig 1D). In CLD with and without compensated cirrhosis, however, MERTK and TYRO3 expressions were sparse (Figs 1D and E, and S1A). Circulatory plasma levels of the AXL ligand GAS6 were significantly elevated in cirrhosis compared with HC, independent of the aetiology. GAS6 increased from Child A to C and correlated with AXL-expressing monocytes (Figs 1F and S1C).

### Circulating AXL-expressing monocytes in patients with advanced cirrhosis indicate diseases severity, complications, and poor outcome

**Figure 2.**
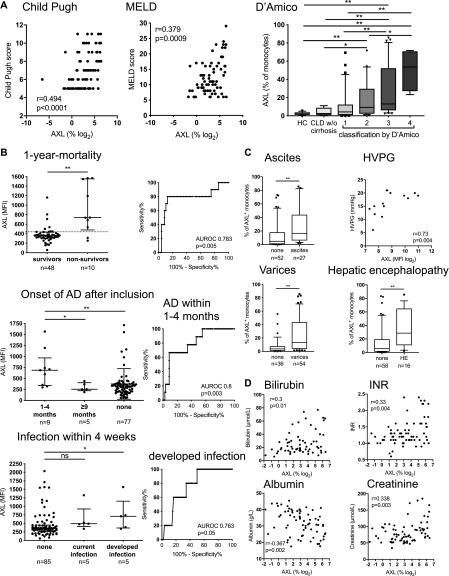
The AXL-expressing monocyte population in patients with cirrhosis in relation to disease severity, complications and prognosis. **(A)** Correlations of AXL-expressing monocytes (%) with Child-Pugh (n = 78) and MELD (n = 73) scores and the classification by D’Amico et al (21). HC, CLD without (w/o) cirrhosis. Box plots showing median/10–90 percentile. **(B)** AXL expression predicted 1-yr mortality (criterion MFI > 440, sensitivity 80%, specificity 79.2%), development of further episodes of AD of cirrhosis within 4 mo (criterion MFI > 362, sensitivity 66.7%, specificity 67.1%), and development of infection over 4 wk (criterion MFI > 389, sensitivity 60%, specificity 65.5%). Median/interquartile range (IQR). **(C, D)** AXL-expressing monocytes in relation to portal hypertension (C: ascites, hepatic venous pressure [HVPG, n = 14], varices, hepatic encephalopathy; D: bilirubin, n = 72; INR, n = 74; albumin, n = 72; and creatinine, n = 75). Median/10–90 percentile. *P < 0.05/**P < 0.01 (Mann–Whitney tests, Spearman correlation coefficient).

**Figure S3.**
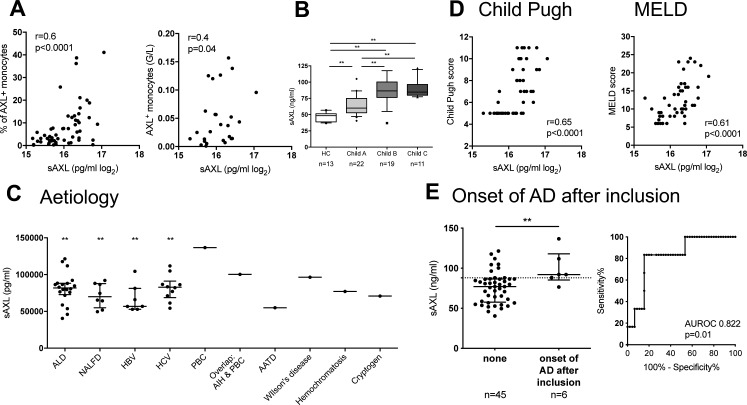
sAXL plasma levels in cirrhosis related to disease severity and acute decompensated cirrhosis. **(A)** Correlation of sAXL levels with AXL-expressing monocytes (n = 64) and absolute numbers (G/L; n = 27). Spearman correlation coefficient. **(B)** sAXL plasma levels (ng/ml) in patients with cirrhosis compared with HCs. Median/10–90 percentile (Mann–Whitney tests). **(C)** sAXL plasma levels in different underlying aetiologies of cirrhosis. Alcoholic liver disease (n = 20); nonalcoholic fatty liver disease (n = 8); hepatitis B virus (n = 7); hepatitis C virus (n = 10); (PBC; n = 1); autoimmune hepatitis & PBC (AIH & PBC; n = 1); alpha-1 antitrypsin deficiency (n = 1); Wilson’s disease (n = 1); hemochromatosis (n = 1); and cryptogenic cirrhosis (n = 1). Median with IQR. Statistical significance levels compared with HC (Mann–Whitney test). **(D)** Correlation of sAXL levels with Child-Pugh (n = 47) and MELD scores (n = 47). Spearman correlation coefficient. **(E)** sAXL predicted the onset of acute decompensated cirrhosis (AD) episodes within 4 mo following inclusion for the criterion sAXL > 88.1 ng/ml (sensitivity 83.3%, specificity 82.2%). Median with IQR. *P < 0.05, **P < 0.01 (Mann–Whitney test).

We next assessed the expansion of AXL-expressing monocytes in relation to clinical parameters, disease severity scores, indicators of complications, and outcome. The proportion of AXL-expressing monocytes strongly correlated with Child-Pugh and model for end-stage liver disease (MELD) scores and the classification of cirrhosis established by D’Amico et al (21) (Fig 2A). AXL-expressing monocytes also correlated with soluble AXL (sAXL) plasma levels. sAXL was significantly elevated in cirrhosis compared with controls, independent of the underlying aetiology, correlated with Child-Pugh and MELD, and predicted the onset of AD episodes within 4 mo following study inclusion (Fig S3A–E).

**Figure S4.**
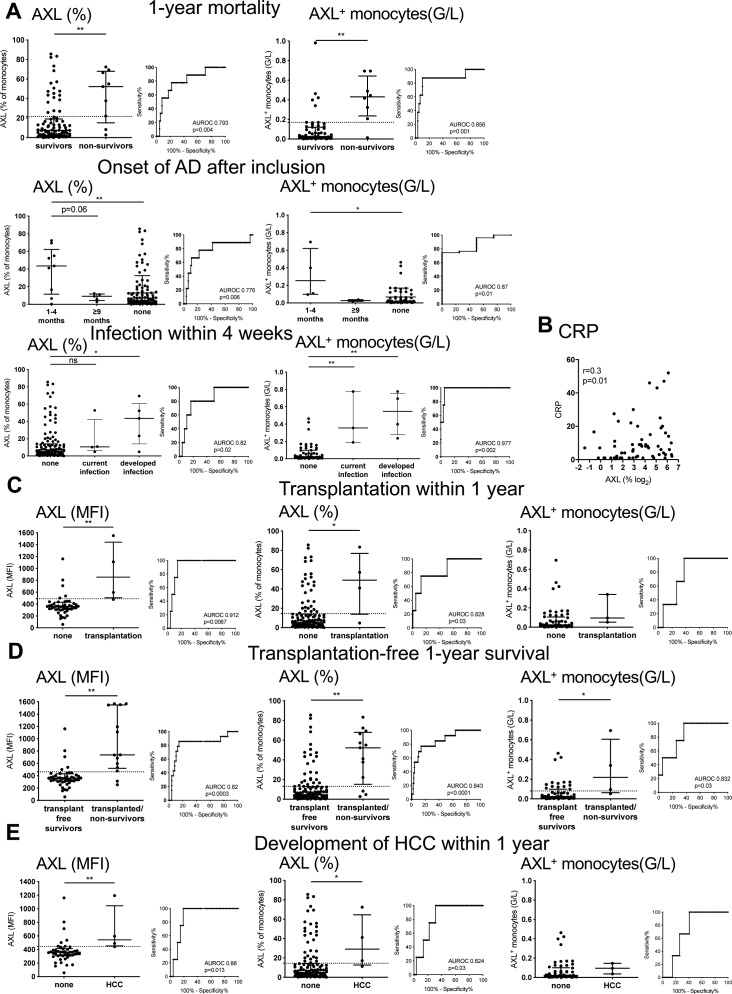
AXL is associated with 1-yr mortality, development of acute decompensated cirrhosis, and inflammation and possibly predicts need for transplantation, transplantation-free 1-yr survival, and development of HCC. **(A)** AXL predicts (A) 1-yr mortality (sensitivity 77.8%, specificity 77.9%, and criterion AXL [%] > 21.6; sensitivity 87.5%, specificity 88.14%, and criterion AXL [G/L] > 0.17), onset of acute decompensated cirrhosis (AD) within 4 mo following inclusion (sensitivity 77.8%, specificity 77.8%, and criterion AXL [%] > 15.8; sensitivity 74.5%, specificity 75%, and criterion AXL [G/L] > 0.097), and development of infection within 4 wk (sensitivity 80%, specificity 79.8%, and criterion AXL [%] > 19.4; sensitivity 100%, specificity 94.6%, and criterion AXL [G/L] > 0.2). Median with IQR (Mann–Whitney tests). **(B)** Correlation of AXL-expressing monocytes with CRP (n = 64). Spearman correlation coefficient. **(C, D, E)** AXL expression on monocytes is associated with the need for transplantation within 1 yr (sensitivity 75%, specificity 85.4%, and criterion AXL MFI > 490; sensitivity 75%, specificity 74.8%, and criterion AXL [%] > 13.6; sensitivity 66.7%, specificity 66.1%, and criterion AXL [G/L] > 0.067), with (D) transplantation-free 1-yr survival (sensitivity 85.7%, specificity 85.4%, and criterion AXL [MFI] > 463.5; sensitivity 76.9%, specificity 76.1%, and criterion AXL [%] > 13.1; sensitivity 75%, specificity 74.5%, and criterion AXL [G/L] > 0.081), and with (E) the development of HCC within 1 yr (sensitivity 75%, specificity 80.1%, and criterion AXL [MFI] > 444; sensitivity 75%, specificity 74.4%, and criterion AXL [%] > 14.5). Median with IQR. *P < 0.05, **P < 0.01 (Mann–Whitney tests).

AXL on monocytes predicted 1-yr mortality with a sensitivity of 80% and specificity of 79.2% for the criterion median fluorescence intensity (MFI) > 440. AXL moreover predicted the onset of AD within 4 mo following inclusion, and the development of infection over the next 4 wk. AXL was also associated with C-reactive protein (CRP) (Figs 2B and S4A, and B). Furthermore, AXL-expressing monocytes were associated with manifestations of portal hypertension (ascites, hepatic venous pressure gradient, varices, hepatic encephalopathy, and renal dysfunction) and correlated with individual parameters of liver function (bilirubin, international normalised ratio [INR], albumin) (Fig 2C and D). High AXL expression on monocytes may albeit small numbers also predict the need for transplantation, transplantation-free 1-yr survival, and development of HCC within 1 yr (Fig S4C–E).

### Phenotype of circulating monocytes in patients with cirrhosis and the AXL-expressing monocyte population

**Figure S5.**
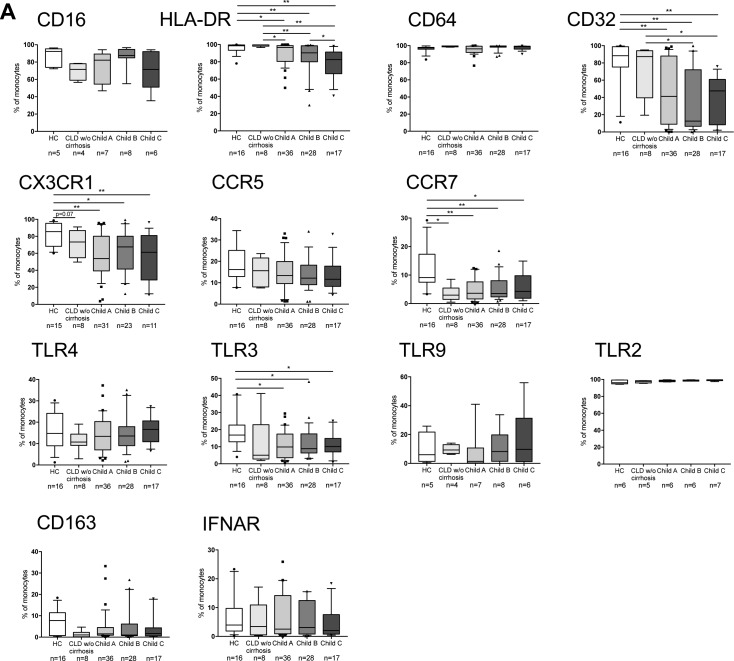
Phenotypic characterisation of circulating monocytes. **(A)** Immunophenotyping of monocytes in HCs, CLD without (w/o) cirrhosis, and cirrhosis: Fcγ-receptors (CD16, CD32, and CD64), MHC class II receptor HLA-DR, chemokine receptors (CX3CR1, CCR5, and CCR7), TLR4, 3, 9, and 2, scavenger receptor CD163, and IFN-α/β receptor IFNAR. Median/10–90 percentile. *P < 0.05, **P < 0.01 (Mann–Whitney tests).

Monocytes from patients with CLD without cirrhosis did not differ phenotypically from HC. Monocytes from patients with cirrhosis, however, showed an HLA-DR_low_ phenotype with decreased expression of Fcγ- and homing receptors (CD32_low_CX3CR1_low_CCR7_low_). HLA-DR expression significantly decreased from Child A to C (Fig S5A).

**Figure 3.**
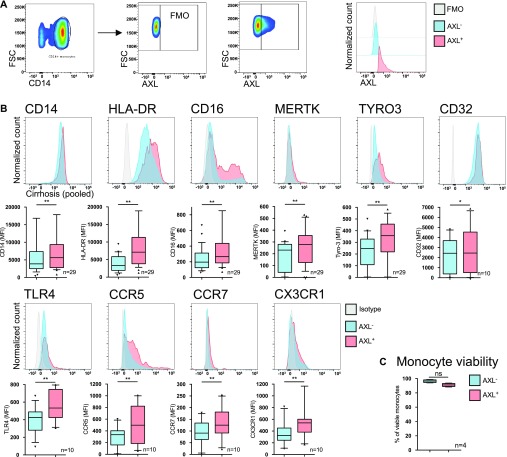
Phenotypic characterisation of the AXL-expressing monocyte subset. **(A)** Gating strategy with representative FACS scatter plots and histograms for AXL expression used to distinguish AXL-expressing (AXL^+^) from AXL-negative (AXL^−^) monocytes. Side scatter (SSC), forward scatter (FSC), fluorescence minus one. **(B)** Immunophenotyping of AXL^+^ and AXL^−^ monocytes in cirrhosis. Glycoprotein CD14, MHC class II receptor HLA-DR, Fcγ-receptors (CD16 and CD32), TAM receptors (MERTK and TYRO3), chemokine receptors (CX3CR1, CCR5, and CCR7), and TLR4. Box plots showing median/10–90 percentile. **(C)** Viability (7-AAD^−^AnnexinV^−^-cells) of AXL^+^-/AXL^−^-monocytes. Median/10–90 percentile. *P < 0.05/**P < 0.01 (Wilcoxon tests).

Within this entire population, the expanded subset of AXL-expressing monocytes (AXL^+^) (Fig 3A) were CD14^+^CD16^high^HLA-DR^high^ indicating a mature monocyte subpopulation with augmented expression of Fcγ-receptor CD32, TLR4, and homing/chemokine receptors (CCR5, CCR7, and CX3CR1) (Fig 3B). There was no difference in viability between AXL^+^- and AXL^−^-monocytes (Fig 3C).

**Figure S6.**
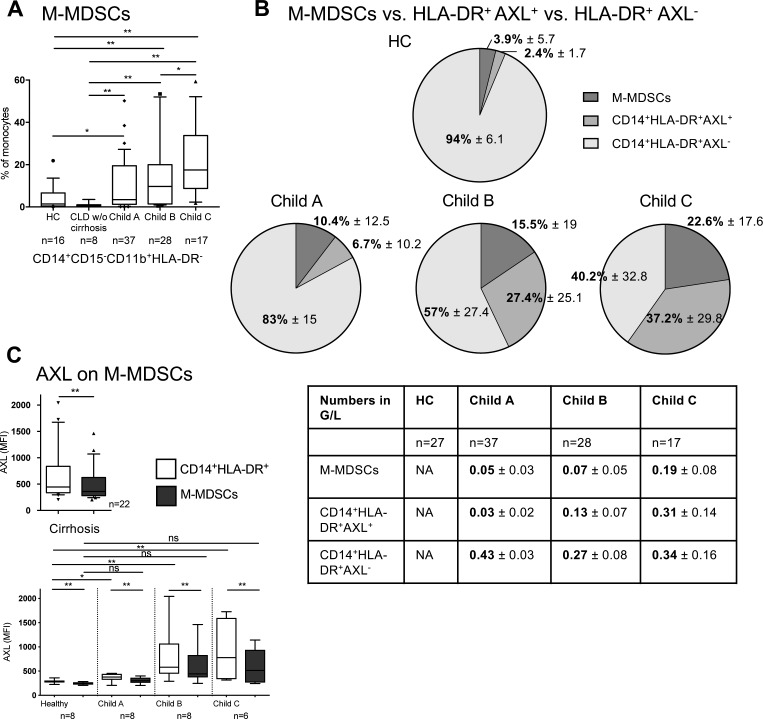
Expansion of immune-regulatory AXL-expressing monocytes and immunosuppressive M-MDSCs in progressing cirrhosis. **(A)** M-MDSCs in patients with stable and advanced cirrhosis compared with patients with CLD without (w/o) cirrhosis and HCs. Median/10–90 percentile (Mann–Whitney tests). **(B)** Distinct monocytic subsets (M-MDSCs, CD14^+^HLA-DR^+^AXL^+^) and “functionally intact” CD14^+^HLA-DR^+^AXL^−^ monocytes (% in pie charts and absolute cell counts in table) with progressing disease severity from Child-Pugh A to C compared with HC represented. Mean with SD. **(C)** AXL expression in M-MDSCs and CD14^+^HLA-DR^+^ monocytic subsets. Median/10–90 percentile. *P < 0.05, **P < 0.01 (Wilcoxon tests).

Importantly, the CD14^+^HLA-DR^+^AXL^+^ immune cell subset detailed here has to be distinguished from the recently identified immunosuppressive M-MDSCs in patients with cirrhosis and ACLF (20), which we observed expanding from Child A to C in our cohort. M-MDSCs were CD14^+^CD15^−^CD11b^+^HLA-DR_low/neg_, as previously defined (22), and expressed lower levels of AXL in comparison with CD14^+^HLA-DR^+^ monocytes (Fig S6A–C).

### AXL-expressing circulating monocytes contribute to impaired innate immune responses and suppression of T cell proliferation while retaining phagocytic capabilities for bacteria ex vivo

To investigate the effect of the expanded CD14^+^HLA-DR^+^AXL^+^ subset on the impaired inflammatory cytokine responses observed in monocytes from patients with cirrhosis we assessed the functional properties of AXL^+^ monocytes ex vivo.

**Figure 4.**
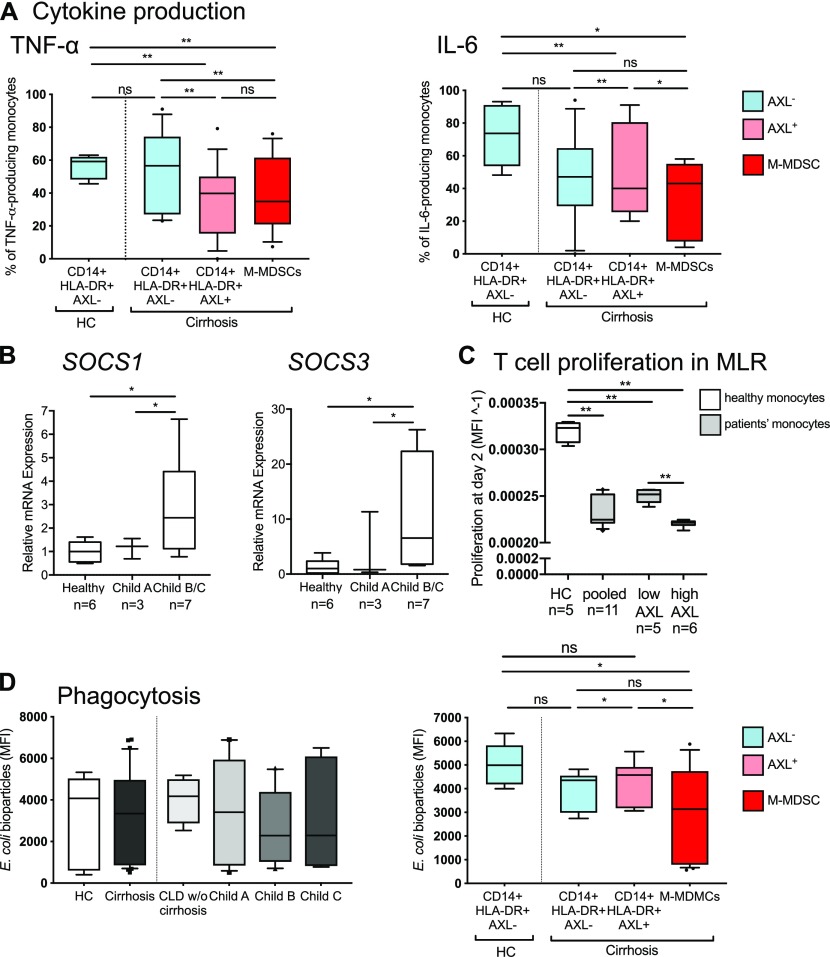
Functional characterisation of AXL-expressing circulating monocytes ex vivo. **(A)** TNF-α and IL-6 production upon LPS treatment of CD14^+^HLA-DR^+^AXL^+^, CD14^+^HLA-DR^+^AXL^−^ monocytes and M-MDSCs from HC and patients with cirrhosis (% of monocytes). **(B)** SOCS1/3 mRNA-expression of monocytes from HC and cirrhosis. **(C)** T cell proliferation in co-culture with monocytes at day 2 in a mixed lymphocyte reaction (HC versus cirrhosis; AXL_low_ versus AXL^high^). Data shown as MFI^−1^ of carboxyfluorescein succinimidyl ester. **(D)** Phagocytosis of *E*. *coli* bioparticles of the entire monocyte population from different patient groups (HC, CLD without [w/o] cirrhosis, cirrhosis, left panel) and CD14^+^HLA-DR^+^AXL^+^, CD14^+^HLA-DR^+^AXL^−^ subsets, and M-MDSCs from HC and patients with cirrhosis. Box plots showing median/10–90 percentile. *P < 0.05/**P < 0.01 (Mann–Whitney, Wilcoxon tests).

**Figure S7.**
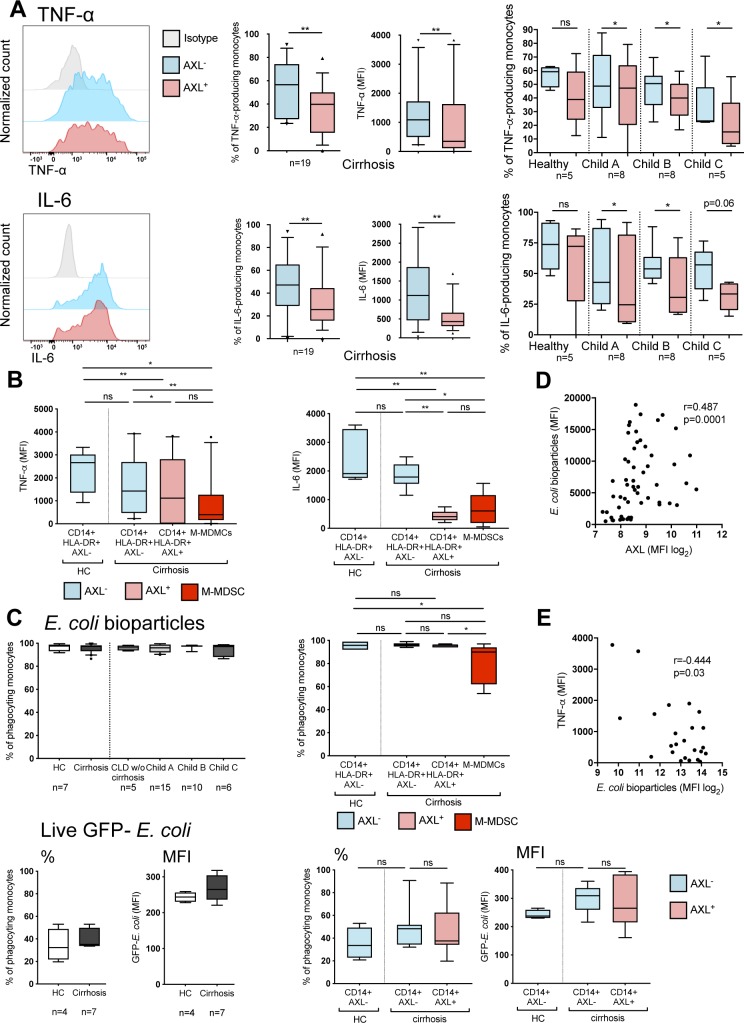
Pro-inflammatory cytokine production in response to LPS, phagocytic capacity of monocytes, and correlations of AXL expression with monocyte function. **(A, B)** TNF-α and IL-6 production (% of monocytes/MFI) upon LPS treatment of monocyte subgroups (AXL^+^-/AXL^−^-monocytes [A]/monocytic myeloid-derived suppressor cells [M-MDSCs; B]) from HCs and patients with cirrhosis at different stages of disease with representative FACS histograms (A). Median/10–90 percentile (Wilcoxon tests). **(C)** Proportion of phagocyting (*E*. *coli*) monocytes (AXL^+^-/AXL^−^-monocytes/M-MDSCs) from HC and patients with cirrhosis. Median/10–90 percentile (Wilcoxon, Mann–Whitney tests). **(D, E)** Correlation of phagocytosis capacity of *E*. *coli* bioparticles with (D) AXL expression (n = 57) and with (E) TNF-α production upon LPS treatment (n = 25). *P < 0.05, **P < 0.01 (Spearman correlation coefficient).

Detailed analyses of the distinct subsets revealed that TNF-α/IL-6 production in response to LPS was decreased in both CD14^+^HLA-DR^+^AXL^+^ monocytes and M-MDSCs when compared with CD14^+^HLA-DR^+^AXL^−^ monocytes from patients with cirrhosis and HC. In detail, TNF-α production decreased from 59.2% (13.8) to 40% (34.6) of monocytes in CD14^+^HLA-DR^+^AXL^−^ from HC versus CD14^+^HLA-DR^+^AXL^+^ from patients (MFI: 2665 [1668] versus 1117 [2812]) and IL-6 from 73.7% (37.3) to 40% (55) (MFI: 1909 [1702] versus 408 [298]; median [interquartile range, IQR]) Figs 4A and S7A, and B). The CD14^+^HLA-DR^+^AXL^−^ population represented the majority of monocytes in HC, indicating it may be regarded “functionally intact” but was sequentially lost in the circulation of patients with progression of cirrhosis (Fig S6B). In line with previous data detailing TAM receptor signalling pathways (16), we observed higher mRNA levels of SOCS1/SOCS3 in monocytes of Child B/C patients, compared with HC (Fig 4B). Our data thus reveals the determination of functional roles of monocyte subsets in a pathophysiological context such as cirrhosis.

We further revealed that AXL expression on monocytes in cirrhosis was associated with inhibition of T cell proliferation, when tested in an allogeneic mixed lymphocyte reaction (Fig 4C).

Ex vivo phagocytic capacity of *Escherichia coli* (*E*. *coli*) bioparticles and live GFP-containing *E*. *coli* by circulating monocytes did not differ between cirrhotic patients and HC. AXL^+^ monocytes showed preserved phagocytosis capacities for *E*. *coli* bioparticles and live *E*. *coli* bacteria, whereas M-MDSCs revealed reduced phagocytosis, when compared with CD14^+^HLA-DR^+^AXL^−^ monocytes from patients with cirrhosis and HC (Figs 4D and S7C).

Considering these observations, the expanded CD14^+^HLA-DR^+^AXL^+^ monocyte population in the circulation of patients with cirrhosis (notably, not existing in healthy subjects) remained functionally phagocytic, but prevented T cell proliferation and inflammation (low TNF-α/IL-6 production) in a presumably SOCS1/3-dependent manner, representing an immune-regulatory “homeostatic” monocyte population expanding during cirrhosis progression.

### AXL overexpression in THP-1 cells attenuates LPS-induced inflammatory cytokine production in vitro

**Figure 5.**
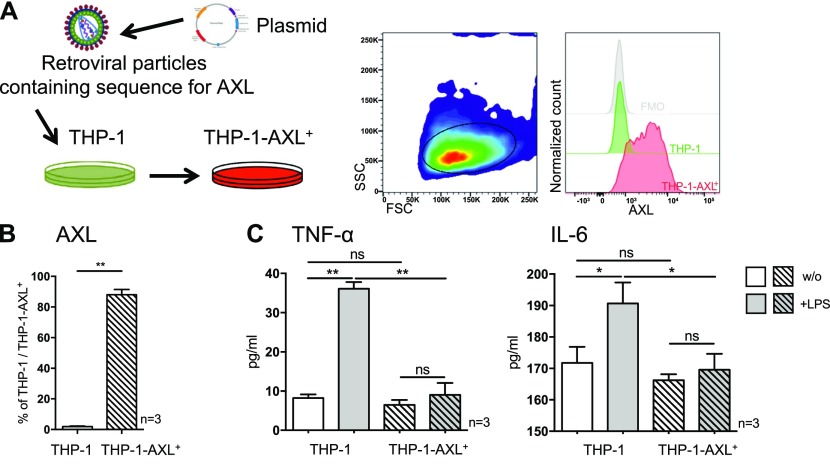
AXL overexpression in THP-1 cells and LPS-induced inflammatory cytokine production in vitro. **(A)** Schematic model of retroviral transduction of THP-1 cells and representative FACS histogram of AXL expression in THP-1-AXL^+^ cells. Side scatter (SSC), forward scatter (FSC). **(B)** AXL expression in THP-1 cells and AXL-expressing THP-1 cells (%). **(C)** TNF-α and IL-6 secretion (pg/ml) in response to LPS in THP-1-AXL^+^ and THP-1 cells. Bar plots showing mean/SD. *P < 0.05/**P < 0.01 (*t* tests).

**Figure S8.**
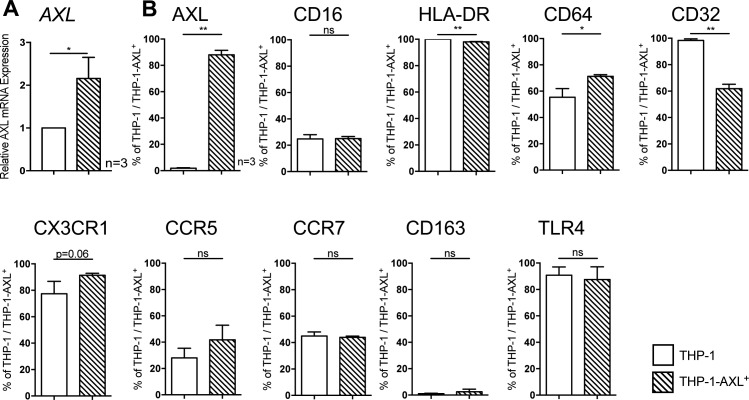
Phenotypic characterisation of monocytic THP-1 and transduced THP-1-AXL^+^ cells. **(A)** Relative AXL mRNA expression in THP-1 compared with THP-1-AXL^+^ cells. **(B)** Difference of protein surface receptors (Fcγ-receptors [CD16, CD32, and CD64], MHC class II receptor HLA-DR, chemokine receptors [CX3CR1, CCR5, and CCR7], TLR4, and scavenger receptor CD163) on THP-1 and THP-1-AXL^+^ cells. Mean % of THP-1/THP-1-AXL^+^ cells with SD. *P < 0.05, **P < 0.01 (*t* tests).

As proof-of-concept for the observations developed above, in vitro, we overexpressed AXL in the monocytic THP-1 cell line using a retroviral system (Fig 5A). Following transduction, AXL mRNA expression (2.2 ± 0.3-fold; Fig S8A) and protein levels (88% THP-1-AXL^+^ cells; Fig 5A and B) were increased. Phenotypic characterisation of the THP-1-AXL^+^ model cell line is illustrated in Fig S8B. Consistent with the observations in patients with cirrhosis ex vivo, AXL-expressing THP-1 cells produced less TNF-α and IL-6 in response to LPS when compared with non-transduced THP-1 cells (Fig 5C).

### Pathogen-associated molecular patterns (PAMPs), cytokines, bacterial uptake, and efferocytosis induce AXL up-regulation on monocytes

**Figure 6.**
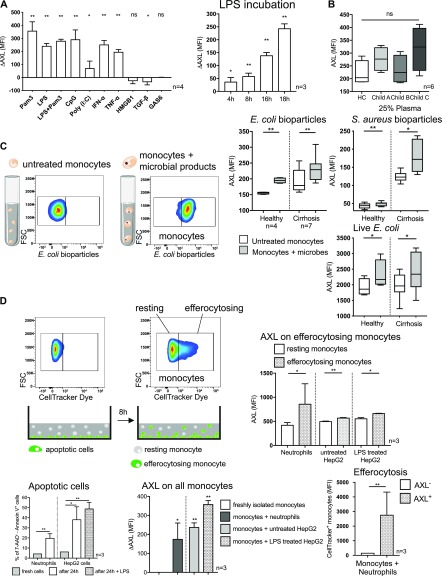
AXL expression on monocytes in response to bacterial and inflammatory stimuli and following phagocytosis and efferocytosis. **(A)** AXL expression after incubation with bacterial/inflammatory stimuli as indicated in vitro for 18 h. Time-dependent effect of LPS on AXL expression. Delta AXL (ΔAXL) MFI shows difference to untreated cells. Bar plots showing mean/SD (*t* tests). **(B)** AXL expression after monocyte incubation in 25% plasma of HCs and patients with cirrhosis for 24 h. **(C)** Representative FACS scatter plots for monocyte phagocytosis of microbial products ex vivo. Forward scatter (FSC). AXL expression after *E*. *coli* and *S*. *aureus* bioparticle uptake (15 min) and live GFP-*E*. *coli* ingestion (60 min) on monocytes from HC and patients with cirrhosis. Box plots showing median/10–90 percentile (Mann–Whitney tests). **(D)** Representative FACS scatter plots for resting (CellTracker^−^) and efferocytosing (CellTracker^+^) monocytes after co-culture with apoptotic cells for 8 h. Apoptosis of neutrophils and HepG2 cells after 24 h. AXL expression after efferocytosis, AXL expression of resting and efferocytosing monocytes, and efferocytosis capacity for neutrophils of AXL^+^-/AXL^−^-monocytes. Bar plots showing mean/SD. *P < 0.05/**P < 0.01 (unpaired/paired *t* tests).

**Figure S9.**
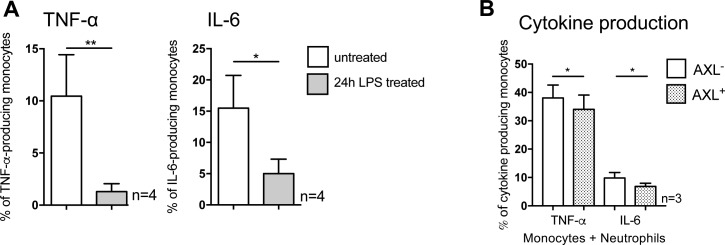
Cytokine responses following up-regulation of AXL. **(A, B)** TNF-α and IL-6 production of healthy monocytes upon LPS treatment (100 ng/ml, 5 h) after 24-h pre-incubation with LPS (which resulted in AXL up-regulation; Fig 6A) compared with monocytes not previously exposed to LPS (A) and after 8-h co-culture with apoptotic neutrophils (which also resulted in AXL up-regulation; Fig 6D; AXL^+^ versus AXL^−^ monocytes) (B). Mean % of monocytes with SD. *P < 0.05, **P < 0.01 (*t* tests).

Next, we sought to understand the mechanisms leading to the expansion of the described immune-regulatory AXL-expressing monocyte population. Pathophysiologically, cirrhosis progression involves development of portal hypertension and subsequent pathologic bacterial translocation facilitates microbial products accessing the systemic circulation (8, 13, 14, 23). Hence, we tested PAMPs and damage-associated molecular patterns (DAMPs) for their ability to modify monocyte differentiation and AXL expression in vitro. Stimulation with bacterial products such as TLR ligands (Pam3SK4, LPS, CpG, and poly (I:C)) significantly up-regulated AXL expression in vitro. Similarly, pro-inflammatory factors (IFN-α and TNF-α) induced AXL up-regulation. In contrast, the DAMP high-mobility group protein B1 (HMGB1), TGF-β, and AXL ligand GAS6 did not induce AXL expression. LPS-induced up-regulation of AXL was time dependent (Fig 6A), and those monocytes produced significantly less TNF-α/IL-6 upon LPS when compared with monocytes without prior LPS exposure (Fig S9A). Notably, monocytes incubated with 25% plasma of cirrhosis patients did not show biologically relevant changes in AXL expression (Fig 6B), suggesting that additional factors are required to generate this subset in vivo.

Phagocytosis is required for efficient clearance of pathogenic microorganisms and initiation of various immune responses. AXL^+^ monocytes exhibited preserved phagocytosis capacities (Figs 4D and S7C) and AXL expression significantly increased on monocytes after phagocytosis of *E*. *coli* and *Staphylococcus aureus* (*S*. *aureus*) bioparticles as well as of live GFP-*E*. *coli* bacteria (Fig 6C). Phagocytosis capacity of *E*. *coli* bioparticles positively correlated with the degree of AXL expression and concurrently negatively with TNF-α production (Fig S7D–E).

Within inflammatory milieus, as prevalent in different compartments of patients with cirrhosis (8), efferocytosis is required to maintain immune homeostasis (24, 25). We, therefore, co-cultured healthy monocytes with neutrophils and HepG2 cells previously labelled with a cytoplasmic cell-tracker and then induced to apoptosis (19, 26). AXL expression on monocytes was up-regulated following co-culture with apoptotic cells. Monocytes that engulfed apoptotic cells (efferocytosing) were characterised by higher AXL expression, compared with monocytes that did not (resting). AXL^+^ monocytes showed higher efferocytosis capacity than AXL^−^ monocytes when co-cultured with apoptotic neutrophils (Fig 6D); following efferocytosis of neutrophils, AXL^+^ monocytes produced less TNF-α/IL-6 upon LPS than AXL^−^ monocytes (Fig S9B), supporting the accumulation of AXL^+^ immune-regulatory monocytes in an inflammatory environment.

### AXL inhibitor BGB324 and metformin restore innate immune responses of monocytes from patients with cirrhosis ex vivo

**Figure 7.**
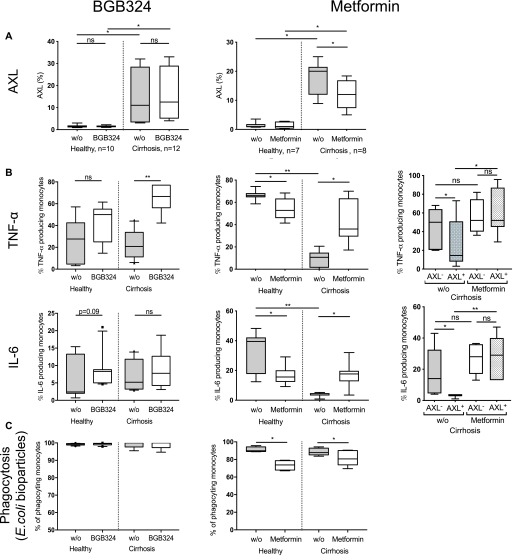
Innate immune responses and phagocytosis capacity of monocytes from patients with cirrhosis after AXL inhibition and down-regulation ex vivo. **(A, B, C)** AXL expression (% of monocytes), (B) TNF-α and IL-6 production in response to LPS (total monocyte population and AXL^+^/AXL^−^-cells), and (C) monocyte phagocytosis capacity of *E*. *coli* bioparticles (%CD14^+^ cells) after small molecule inhibitor BGB324 and metformin treatment compared with untreated cells (w/o) in HCs and patients with cirrhosis. Box plots showing median/10–90 percentile. *P < 0.05/**P < 0.01 (Mann–Whitney, Wilcoxon tests).

**Figure S10.**
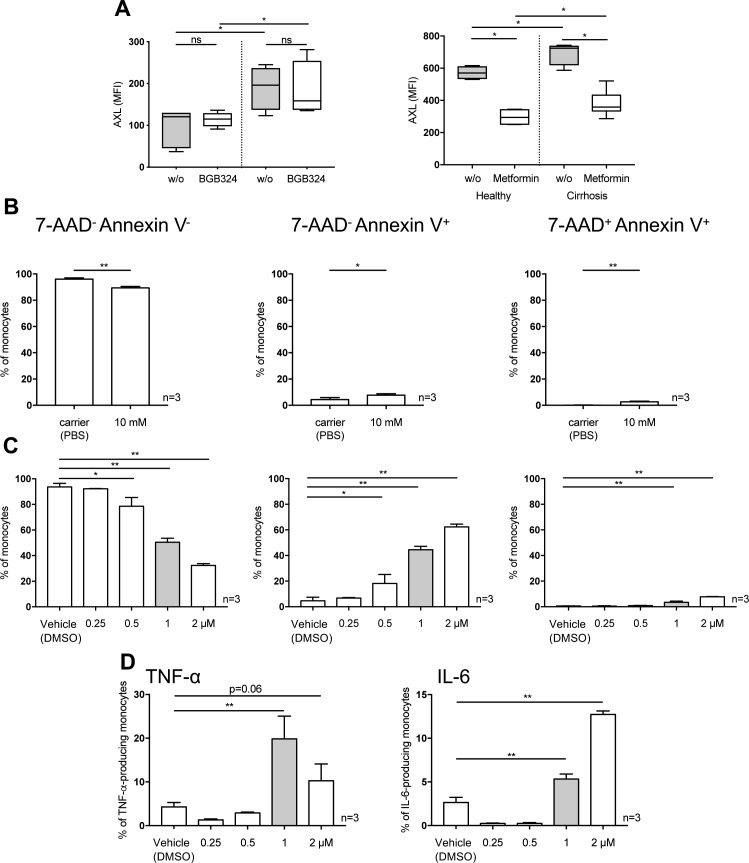
AXL expression, viability, and cytokine expression after BGB324 and metformin treatment. **(A)** AXL expression (MFI) after BGB324 and metformin treatment compared with untreated (w/o) cells in HCs and patients with cirrhosis. Median/10–90 percentile (Mann–Whitney tests). **(B, C)** Healthy monocytes were treated with 10 mM of metformin (24 h) (B), 0.25, 0.5, 1, and 2 μM of BGB324 (24 h) (C) and proportion of viable (7-AAD^−^-Annexin V^−^), apoptotic (7-AAD^−^-Annexin V^+^), and dead (7-AAD^+^-Annexin V^−^) monocytes was assessed. **(D)** TNF-α/IL-6 production was assessed in response to LPS (100 ng/ml, 5 h) after different doses of BGB324. DMSO. Mean % of monocytes with SD. *P < 0.05, **P < 0.01 (*t* tests).

Given the distinct immune-regulatory functions of the AXL^+^ monocyte population in patients with cirrhosis and its association with disease severity and infection, we questioned whether inhibition or down-regulation of AXL would reverse the anti-inflammatory properties. BGB324 is a selective small molecule inhibitor of AXL previously tested in clinical studies (27). Metformin, a well-known antidiabetic drug, was previously described to down-regulate AXL expression (28) and to regulate the AXL signalling cascade in the context of cancer (29, 30). Metformin-induced down-regulation of AXL was confirmed in monocytes from patients with cirrhosis ex vivo here. Treatment with BGB324 did not affect AXL expression (Figs 7A and S10A). Both, BGB324 (1 μM) and metformin (10 mM) treatment restored LPS-induced TNF-α production of monocytes from patients with cirrhosis ex vivo. When comparing AXL^+^ with AXL^−^ monocyte populations from patients with cirrhosis following metformin treatment, cytokine production was enhanced in AXL^+^ but not AXL^−^ cells (Fig 7B). Viability of monocytes after metformin treatment was marginally reduced (Fig S10B). Whereas phagocytosis capacity of *E*. *coli* bioparticles was preserved after BGB324 administration, it decreased upon metformin treatment (Fig 7C).

## Discussion

In this work, we detail the characteristics of circulating monocytes in patients suffering from cirrhosis at different stages of disease without signs of AD. We newly describe the expansion of an AXL-expressing immune-regulatory monocyte subset (CD14^+^HLA-DR^+^AXL^+^) along cirrhosis progression and its close association with disease severity, infection susceptibility, development of AD, and prognosis. The AXL-expressing monocyte generation was linked to the abundance of PAMPs and cytokines, phagocytosis, and efferocytosis in the context of recurrent inflammation. Our findings substantially add to the understanding of the pathophysiology of immuneparesis in cirrhosis and identify a potential biomarker and immunotherapeutic target.

Although we and others have previously focused on impaired innate immune responses after the onset of AD/ACLF, when infection susceptibility is highest (10, 12, 18, 20), it is barely known when and under which circumstances innate immune dysfunction occurs and infection susceptibility emerges during progression of cirrhosis and portal hypertension. Previous studies addressing phenotype and function of classical (CD14^+^CD16^−^) and nonclassical (CD14^+^CD16^+^) monocyte subsets in CLD showed inconsistent data regarding cytokine production and phagocytosis (31, 32, 33). We previously showed that inflammatory cytokine production was depressed not only in AD/ACLF but also in stable cirrhosis (18), whereas the underlying mechanism remained unexplained.

Here, we demonstrate the accumulation of circulating CD14^+^HLA-DR^+^AXL^+^ monocytes with attenuated innate immune functions, that is, decreased inflammatory cytokine production (TNF-α/IL-6) and T cell activation, along disease progression of cirrhosis in compensated and chronically decompensated patients. Although dysfunctional monocytes were rarely encountered in Child A, their number substantially expanded in advanced stages (Child B/C) and displaced functionally intact monocytes. The findings were irrespective of the underlying aetiology. Monocyte functions were preserved in patients with CLD without cirrhosis (F ≤ 3). Follow-up data of individual patients showed an evolution of AXL-expressing monocytes in parallel with disease severity scores. However, this requires further evaluation in larger prospective longitudinal studies.

AXL is a member of TAM receptors that mainly function as inhibitors of TLR- and cytokine receptor–mediated monocyte/macrophage activation and promoters of apoptotic cell removal (15, 16). Loss of AXL expression on antigen-presenting cells has been linked to autoimmunity (15). TAM receptors are differentially expressed and exhibit distinct expression, regulation, and activity under specific conditions (34, 35). Although CD14^+^HLA-DR^+^AXL^+^ cells accumulated with worsening stages of cirrhosis, they disappeared upon acute hepatic decompensation. By contrast, CD14^+^HLA-DR^+^MERTK^+^ monocytes remained undetectable in stable cirrhosis and emerged upon AD (18). In addition, CD14^+^HLA-DR^+^MERTK^+^ cells were abundant in the circulation and the liver in acute liver failure where they were characterised as resolution-type monocytes/macrophages (19). This underlines the distinct and counter-regulatory roles of AXL and MERTK in different phases of cirrhosis and inflammation. Our findings in a human disease verify previous murine data that identified differential and reciprocal expression and function between AXL and MERTK on BMDMs/BMDCs (34). The authors described both receptors as phagocytic mediators in vitro, whereas MERTK expression was induced by tolerogenic stimuli and induced tolerance, and AXL was induced by inflammatory stimuli and acted in the feedback inhibition of inflammation (34). The underlying differential signalling mechanisms need to be addressed in future investigations.

Extensive characterisation of the AXL-expressing monocyte population revealed an immune-regulatory subset that emerged in the circulation likely to maintain immune homoeostasis despite rising inflammatory signals during progression of cirrhosis. AXL-expressing monocytes were characterised by increased HLA-DR, CD16 and chemokine-receptor expression, enhanced clearance of apoptotic cells, increased expression of Fcγ-receptor, and preserved phagocytosis of *E*. *coli*, but attenuated T cell activation and secretion of pro-inflammatory cytokines (TNF-α/IL-6) after microbial challenge presumably in a SOCS1/3-dependent manner, as previously proposed (16). Although phagocytosis of pathogens represents the first line of defence when an organism encounters microbes, clearance of apoptotic cells is required for immune homeostasis during inflammation. Our findings suggest that AXL-expressing monocytes may expand during cirrhosis and progressive portal hypertension in response to the uptake of pathogens and bacterial products in the setting of pathologic bacterial translocation (8, 13, 14, 23), and to clear apoptotic cell debris accumulating in response to chronic inflammation (15, 16). Concurrently, excessive systemic inflammatory responses are inhibited.

Similar to our findings, a recent study described AXL-expressing murine airway macrophages at homeostatic conditions, which increased after influenza infection, thereby preventing excessive tissue inflammation through efferocytosis (35). AXL expression was critical for functional compartmentalisation, as it was not present on interstitial lung macrophages (35). We propose that in cirrhosis, AXL may be operative in settings where the nature of injury is driven by excessive pro-inflammatory responses, as present in diverse underlying aetiologies.

This newly described immune-regulatory, AXL-expressing monocyte population must be clearly distinguished from another recently discovered immunosuppressive cell subset that accumulated in the circulation in stages of AD: M-MDSCs were characterised by suppression of T cell activation, pathogen uptake, and TLR-elicited pro-inflammatory responses to microbial challenge (20). Here, we observed that M-MDSCs started to emerge also in patients with cirrhosis without signs of AD. Considering the reduced inflammatory responses to microbial challenge of both CD14^+^HLA-DR^+^AXL^+^- and M-MDSCs compared with functionally intact CD14^+^HLA-DR^+^AXL^−^ cells in relation to their abundance in the circulation, we propose that these populations together largely explain the depressed innate immune responses of the entire monocytic population at these stages.

Having observed AXL up-regulation on circulatory monocytes in relation to portal hypertension, we hypothesized an underlying mechanism involving pathologic bacterial translocation leading to the abundance of bacterial products, PAMPs (8, 13, 14, 23), and subsequent chronic systemic inflammation (8, 23). At the same time, chronic liver injury leads to release of DAMPs (36). Indeed, we were able to generate AXL-expressing monocytes with dampened innate immune responses by stimulation with selected TLR ligands and pro-inflammatory factors in vitro. These findings coincide with previous data showing AXL up-regulation on murine BMDMs (34, 35) and peritoneal macrophages (35) upon stimulation with inflammatory stimuli (34, 35). In contrast to M-MDSCs, generated in vitro by culturing monocytes in ACLF plasma (20), inflammatory factors in plasma of patients with cirrhosis were necessary, but insufficient to induce AXL up-regulation alone. Our data support the hypothesis that efferocytosis and phagocytosis of bacteria in the circulation are further required to enhance AXL up-regulation on monocytes. The stimulatory effect of pathogen uptake on AXL expression is novel and may explain high AXL expression on circulating monocytes in conditions where pathogens and their products become abundant because of pathologic bacterial translocation such as cirrhosis. TAM receptor activation after efferocytosis had previously been shown on murine BMDMs/BMDCs (34).

Dissecting the complexity of differential monocyte differentiation and activation of effector pathways of particular TAM receptors on monocytes at different stages of cirrhosis will be subject to future investigations, including the use of unbiased large-scale techniques. In a multisystem disorder such as cirrhosis, additional compartments such as the liver, but also the gut, the portal circulation, the peritoneum, and potentially others and their tissue-specific immune systems play crucial roles in the pathophysiology of the underlying immuneparesis. It is the aim of our subsequent investigations to detail the differentiation and immune function of tissue-specific myeloid cells in these compartments, in particular in respect to the immune-regulatory role of TAM receptors.

Moreover, by ex vivo proof of principle experiments treating monocytes from cirrhotic patients with the highly specific AXL inhibitor BGB324 and metformin, which was previously described to target and down-regulate AXL (28), innate immune responses were significantly enhanced, suggesting AXL as potential immunotherapeutic target to augment defence against infections. Whereas BGB324 did not negatively affect phagocytic capabilities, metformin did.

BGB324 was originally developed for cancer treatment and is currently tested in clinical Phase Ib/II trials for patients with aggressive and metastatic cancers (37). Interestingly, other studies have examined BGB324 as an anti-fibrotic agent. GAS6/AXL pathways were associated with fibrogenesis in CLD (38) and idiopathic pulmonary fibrosis (39), respectively, and were reversed by BGB324. Multi-tyrosine kinase inhibitors, including AXL, are known for their diverse antitumour effects and are tested in phase III clinical trials for advanced HCC (40). Distinct AXL blockage impacts on tumour progression through immune surveillance by AXL-expressing immune cells and anti-proliferative effect on AXL-expressing tumour cells (37, 40). AXL inhibition by BGB324 may, thus, represent a promising concept with anti-fibrotic, immune-stimulatory, and also anti-tumour effects.

Whereas previous studies described anti-inflammatory, presumably AXL-independent properties of metformin on myeloid cells (41), we observed enhanced immune responses of AXL-expressing monocytes from cirrhotic patients after metformin treatment. Metformin, conventionally used as anti-diabetic drug, exerts various pleiotropic effects acting via diverse downstream signalling pathways (42) and has been reported to be associated with reduced HCC incidence (43) and reduced portal hypertension in cirrhosis models (44). Further studies hint at a potential regulatory effect of metformin on the AXL cascade in the context of cancer (29, 30). As an inexpensive, well-established drug, metformin may represent an interesting immunomodulatory treatment option for patients with cirrhosis and no signs of AD, when AXL-expressing monocytes are frequent and the risk for metformin-associated lactic acidosis is low. Our data are suggestive to further investigate the potential significance of metformin in this context and its underlying signalling mechanism.

As these substances were only tested ex vivo here, subsequent in vivo studies in rodent models are required to systematically investigate target- and off-target effects such as auto-immunity or uncontrolled inflammation. We showed previously that inhibition of MERTK on monocytes of AD/ACLF patients reversed innate immune dysfunction (18). Given the distinct and reciprocal expression profiles of AXL and MERTK in cirrhosis, it further needs to be addressed which receptor to target at which stage of disease and in which compartment.

Finally, strong correlations of AXL expression on monocytes with disease severity and prognosis, that is, i.e. development of infection, episodes of AD, and 1-yr mortality underline its clinical significance. Two recent studies suggested sAXL as a serum biomarker for advanced liver fibrosis, cirrhosis, and HCC (45, 46). Here, we observed strong correlations of AXL-expressing monocytes with the shed receptor sAXL and also liver disease severity scores. Based on our findings, the number of AXL-expressing monocytes in blood count may represent a prognostic biomarker for immuneparesis and cirrhosis and validates further evaluation.

In conclusion, the number of AXL-expressing immune-regulatory monocytes in the circulation of patients with cirrhosis indicated disease severity, immuneparesis, infection susceptibility, AD, and mortality. CD14^+^HLA-DR^+^AXL^+^ monocytes were expanded upon PAMP and cytokine exposure, pathogen,- and apoptotic cell uptake and hallmarked by preserved phagocytosis and enhanced efferocytosis but reduced cytokine production and T cell activation, implying a role in immune homeostasis in a condition defined by pathologic bacterial translocation and recurrent inflammation. Immunotherapeutic modulation of AXL may represent an option deserving evaluation to augment immune responses and reduce infection susceptibility, morbidity, and mortality in cirrhosis.

## Materials and Methods

### Patients and sampling

A cohort of 96 patients with cirrhosis was identified at the Cantonal Hospital St. Gallen and the University Hospital Basel, Switzerland, between January 2016 and May 2019. Patients were recruited during consultations (Child-Pugh A [n = 36], B [n = 35], C [n = 17]), respectively, categorised according to Child-Pugh and European Association for the Study of the Liver - Chronic Liver Failure (EASL-CLIF) Consortium scores (47). We included HC (n = 27), patients with CLD without cirrhosis (n = 8; Metavir F ≤ 3), and patients with AD within 24 h following hospital admission (n = 8) as comparators. Healthy volunteers from the regions St. Gallen and Basel, Switzerland, were matched by age and sex and served as HCs. Patients’ assent was obtained by the patients’ nominated next of kin if they were unable to provide informed consent themselves. Cirrhosis was diagnosed by liver biopsy (n = 92, 95.8%) or clinical presentation with typical ultrasound (n = 4, 4.2%). Exclusion criteria for patients were age younger than 18 yr and evidence of metastatic malignancies (including HCC). Five patients with non-metastatic malignancies were included (HCC, Barcelona Clinic Liver Cancer staging system stages A–B [n = 3]; breast cancer, pT1b, pN0, and M0 [n = 1]; prostate carcinoma, Gleason score 7a [n = 1]). Five patients included with AD had infection at inclusion (spontaneous bacterial peritonitis [n = 3]; spontaneous bacterial peritonitis and urinary tract infection [n = 2]) and six patients included were on immunosuppressive therapy (steroids for AD [n = 3]/adrenal insufficiency [n = 1]/allergy [n = 1]; azathioprine for autoimmune hepatitis [n = 2]). Blood specimens were obtained for ex vivo analysis of monocyte differentiation and function, excessive plasma/serum, and PBMCs were stored. Patients were followed-up for 1 yr for adverse events (infection, development of AD after inclusion, mortality, transplantation, and HCC). Evidence of culture-positive/negative infection was documented. The study had been approved by the local ethics committees (EKSG 15/074/EKNZ 2015-308) and recorded in the clinical trial register ClinicalTrials.gov (identifier: NCT04116242) and Swiss National Clinical Trials Portal (SNCTP000003482).

### Clinical, haematologic, and biochemical parameters

Routine clinical and laboratory parameters obtained by the clinician such as full blood count, CRP, INR, liver, and renal function tests and other variables were entered prospectively into a database. Differential blood count at the sites was performed using Sysmex XE differential analyser (Sysmex Europe GmbH) (Cantonal Hospital St. Gallen) and ADVIA 2120i hematology systems (Siemens Healthineers) (University Hospital Basel).

### Monocyte isolation

Monocytes were isolated from PBMCs using CD14 MicroBeads or Pan Monocyte Isolation Kit (Miltenyi Biotec) as previously described (18). Purity of monocytes was assessed by flow cytometry.

### Flow cytometry–based phenotyping of monocytes, assessment of intracellular cytokine responses to LPS stimulation, and viability assay

**Figure S11.**
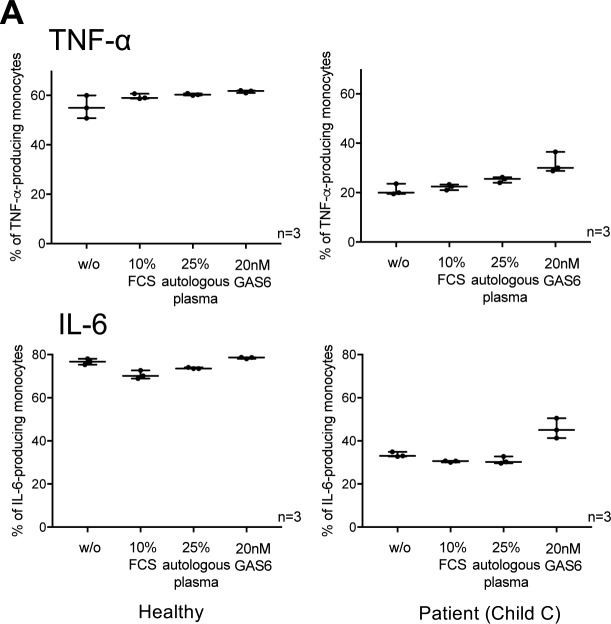
Effect of GAS6 on cytokine production upon LPS treatment. **(A)** Differences of TNF-α/IL-6 production of healthy monocytes and monocytes from patients (Child C) in response to LPS (100 ng/ml, 5 h) after the addition of GAS6 (20 nM) and other complements (10% FCS, 25% autologous plasma) to X-VIVO culture medium.

Phenotyping of monocytes from blood and isolated PBMCs and measurement of inflammatory cytokine production in response to LPS was undertaken using flow cytometry as previously described (18). Antibodies against CD14, CD16, CD163, CD64, CD11b, chemokine receptor (CCR)5, CCR7 (BD Biosciences), CD32, CX3CR1, TLR2, TLR9 (eBioscience), TLR3 (Invitrogen), HLA-DR, CD15, TLR4, TNF-α, IL-6 (BioLegend), TYRO3, AXL, MERTK, and IFNAR (R&D Systems) were purchased from the indicated companies. In addition to ex vivo phenotyping, TNF-α and IL-6 levels were determined after a 5 h incubation of PBMCs with LPS (100 ng/ml) (Invivogen) in X- VIVO medium without complements (Fig S11; Lonza) in a 37°C, 5% CO_2_ environment. The Cells were subsequently acquired on BD FACS Canto or BD LSR Fortessa. Flow cytometric gating strategy for circulating monocytes using whole blood or PBMCs was applied as described in reference 48. Flow cytometry data were analysed using FlowJo software (V.10.4.2; Ashland). Results are expressed as the percentage of positive cells and/or MFI.

Cell viability was determined using the Annexin V Apoptosis Detection Kit I (including 7-AAD) according to manufacturer’s protocols (BD Biosciences).

### Formula for calculating absolute numbers of TYRO3/AXL/MERTK–expressing cells and M-MDSCs

Absolute cell numbers were calculated with the formula: *Frequency of TYRO3/AXL/MERTK-expressing monocytes or M-MDSCs* (% *defined by flow cytometry*) × *monocyte count* (*G/L*).

### sAXL, AXL ligands, and cytokines

sAXL (Abcam), GAS6 (Abnova), IL-6, and TNF-α (R&D Systems) were measured using ELISA in plasma or cell culture supernatants as previously described (18).

### Mixed lymphocyte reaction

Monocytes from HCs and patients with cirrhosis expressing high (>20%/450 MFI) versus low (<7.5%/300 MFI) AXL levels were isolated using the Pan Monocyte Isolation Kit (Miltenyi Biotec) and co-cultured with allogeneic CD3^+^ T cells from a different healthy donor, isolated using the Pan T Cell Isolation Kit (Miltenyi Biotec), in a 1:1 ratio. T cell stimulation was induced with anti-CD2/CD3/CD28 beads (T cell Activation/Expansion Kit; Miltenyi Biotec) as previously described (20). T cells were stained with carboxyfluorescein succinimidyl ester at day 0. Proliferation was assessed at day 2 of co-culture by flow cytometry.

### Generation of THP-1 cells stably expressing AXL

pWZL-Neo-Myr-Flag-AXL vector was a kind gift from Hahn’s laboratory (#20428; Addgene). Packaging plasmids pUMVC and pMD2.G (a gift from Weinberg’s and Trono’s laboratories; #8449 and #12259; Addgene) were used for the production of the retrovirus. THP-1 cells were transduced with pWZL-Neo-Myr-Flag-AXL vector, the cells were selected by G418 (Sigma-Aldrich), and THP-1-AXL^+^ cells with stably introduced pWZL-Neo-Myr-Flag-AXL were subcloned using MethoCult (StemCell Technologies). The clone with highest AXL expression (for purity see Fig S8) was chosen for phenotypic characterisation by flow cytometry, gene expression analysis by quantitative RT-PCR, and LPS-induced cytokine measurement by ELISA. THP-1 and THP-1-AXL^+^ cell lines were cultivated in Roswell Park Memorial Institute 1640 medium (RPMI 1640) (Sigma-Aldrich) supplemented with 10% heat-inactivated FBS, 100 μg/ml streptomycin, and 100 U/ml penicillin (Sigma-Aldrich).

### Quantitative RT-PCR

Total RNA of isolated monocytes from patients with cirrhosis, HCs, and THP-1/THP-1-AXL^+^ cells in cell culture was isolated using RNeasy Mini Kit (QIAGEN) and reversely transcribed into cDNA using High Capacity cDNA Reverse Transcription Kit (Applied Biosystems/Thermo Fisher Scientific). qRT-PCR was performed with 400 ng of cDNA using LightCycler 480 SYBR Green I Master Mix (Roche). Commercial primers for AXL (Hs_AXL_1_SG QuantiTect Primer Assay) were purchased from QIAGEN. Sequences (5′-3′) of primers are as follows: SOCS1 forward: CCC CTT CTG TAG GAT GGT AGC A; reverse: TGC TGT GGA GAC TGC ATT GTC and SOCS3 forward: ATG GTC ACC CAC AGC AAG TT; reverse: TCA CTG CGC TCC AGT AGA AG. GAPDH was used as endogenous control as previously described (35). qRT-PCR was performed according to the manufacturer’s recommendations on QuantStudio Real-Time PCR (Applied Biosystems/Thermo Fisher Scientific).

### Whole blood phagocytosis assay

Whole blood was incubated with pHrodo *E*. *coli* Red BioParticles (Phagocytosis Kit for Flow Cytometry from Invitrogen/Thermo Fisher Scientific) and live *E*. *coli* NovaBlue carrying the gfp-mut2 encoding plasmid pCD353, which expresses a prokaryotic variant of GFP controlled by a lactac promoter as described in (49) (a kind gift of Prof Dr C Dehio, University of Basel). 5 μl of *E*. *coli* Red BioParticles were added for 15 min at 37°C to 100 μl of whole blood and processed as previously described (20). Blood with bioparticles was stained with antibodies against CD14, CD16, HLA-DR, AXL, and CD15, and CD3, CD19, and CD56 (BD Biosciences) and acquired on the flow cytometer. *E*. *coli* bacteria were freshly grown on LB Agar plates supplemented with kanamycin (50 μg/ml; Sigma-Aldrich) and incubated overnight at 37°C. A single colony was picked and grown in LB medium supplemented with kanamycin (50 μg/ml) and IPTG (1 mM; Sigma-Aldrich) for GFP induction at 37°C until early logarithmic growth (OD600 = 0.5–0.6) was reached. After the incubation period, bacteria (1 × 10^9^ bacteria) were centrifuged at 3000*g* for 5 min at 4°C, resuspended in 1 ml PBS, and used immediately. 5 × 10^7^ of GFP-containing *E*. *coli* were added for 60 min at 37°C to 100 μl of whole blood and processed as previously described (20). Blood with GFP-containing *E*. *coli* was stained with antibodies against CD14, CD16, and AXL and acquired on the flow cytometer. The rate of phagocytosis was obtained by the proportion of GFP positive monocytes.

### In vitro inhibition of AXL

A small-molecule inhibitor of AXL, BGB324 (Selleck Chemicals), and metformin (Stemcell Technologies) were used. Selectivity and mechanism of BGB324 were described previously (27, 38). Metformin was previously described to suppress AXL expression at a concentration of 10 mM (28). We used 0.5 × 10^6^ PBMCs from HCs and patients per well on a 48-well plate and cultured them in X-VIVO medium (Lonza) containing 10% FBS in a 37°C, 5% CO_2_ environment. The cells were treated with 1 μM BGB324/10 mM metformin or dimethyl sulfoxide/PBS for 24 h, harvested, and washed two times with PBS before the assessment of inflammatory cytokine production in response to LPS (100 ng/ml, 5 h), phagocytosis capacity, and viability of monocytes by flow cytometry. For the assessment of monocyte phagocytosis from isolated PBMCs in vitro, the harvested cells were incubated with pHrodo *E*. *coli* Red BioParticles (Invitrogen/Thermo Fisher Scientific) for 60 min, processed, and assessed by flow cytometry as previously described (20). The optimal dose of 1 μM BGB324 was initially defined by a dose finding experiment assessing cytokine production of monocytes in response to LPS (100 ng/ml, 5 h). Cell viability using Annexin V (BD Biosciences) was assessed after BGB324 and metformin treatment (Fig S10B–D).

### In vitro models for the generation of AXL-expressing cells

1 × 10^6^ PBMCs per well were cultured on 24-well plates in 1 ml X-VIVO medium (Lonza) in a 37°C, 5% CO_2_ environment. Cells were stimulated with or without LPS 100 ng/ml (4, 8, 16, 18, 24 h), Pam3CSK4 5 μg/ml, CpG 10 μg/ml, poly(I:C) 10 μg/ml (Invivogen), IFN-α 250 U/ml (Roche), TNF-α 250 U/ml, GAS6 20 nM (R&D Systems), HMGB1 20 ng/ml (Sigma-Aldrich), and TGF-β 2 ng/ml (PeproTech) for 18 h. The cells were harvested and subjected to immunophenotyping, intracellular staining of cytokine production in response to LPS and viability assays using flow cytometry as described before.

For the experiments incubating healthy monocytes in plasma from HC (n = 4) and patients with cirrhosis (n = 4), 1 × 10^6^ CD14^+^ cells were cultured in a 24-well plate for 24 h in X-VIVO medium (Lonza) containing 25% of the indicated plasma in a 37°C, 5% CO_2_ environment. Subsequently, a fraction of these cells was phenotyped and the remainder was transferred to fresh medium for the assessment of LPS-stimulated TNF-α/IL6 production and phagocytosis capacity of monocytes by flow cytometry as detailed before.

For the experiments measuring AXL-expressing monocytes after treatment of bacteria, whole blood was incubated with pHrodo *E*. *coli* Red BioParticles, pHrodo *S*. *aureus* Red BioParticles (Invitrogen/Thermo Fisher Scientific) for 15 min, and with live GFP-containing *E*. *coli* bacteria for 60 min and processed as described above.

### Efferocytosis assay

The experimental design was adapted from Zizzo et al (26) and Triantafyllou et al (19). Human neutrophils were isolated using PolymorphPrep (Axis-Shield) by density-gradient centrifugation according to the manufacturer’s protocols, re-suspended at 1 × 10^6^ cells/ml in RPMI-1640 (Sigma-Aldrich) containing 10% FBS (complete medium), labelled with CellTracker Violet BMQC (5 μM in serum-free medium, 45 min, 37°C, dark; Life Technologies, Thermo Fisher Scientific), and incubated for 20 h (37°C in 5% CO_2_) in 300 μl complete RPMI-1640 in 24-well plates. HepG2 cells were seeded at 0.4 × 10^6^ cells/ml in 24-well plates, labelled with CellTracker Violet BMQC as described above, and incubated with LPS (1 μg/ml) for 18 h (37°C in 5% CO_2_) in 300 μl complete RPMI-1640. After the incubation period, percentage of apoptotic neutrophils and HepG2 cells in culture was determined using Annexin V Apoptosis Detection Kit I according to the manufacturer’s protocols (BD Biosciences). Neutrophils and HepG2 cells were re-suspended in the wells and healthy monocytes were added to apoptotic cells (1:4 monocytes to apoptotic cells ratio) for 8 h in 1 ml fresh complete RPMI-1640 (37°C in 5% CO_2_). The cells were harvested and washed two times in PBS and subjected to immunophenotyping, intracellular staining of cytokine production in response to LPS (100 ng/ml, 5 h), and viability assays as described above. The rate of efferocytosis was obtained by the proportion of CellTracker-positive monocytes.

### Statistical analyses

Statistical evaluation was performed in GraphPad Prism v.7.0a (GraphPad Software). *P* < 0.05 values were considered statistically significant. Data are shown as box and whiskers or scatter dot plots and expressed as median with 10–90 percentile, unless otherwise specified. For data that did not follow a normal distribution, significance of differences was tested using Mann–Whitney or Wilcoxon tests. Spearman correlation coefficients and area under the receiver operating characteristic curve were calculated. Normally distributed data were compared using paired or unpaired *t*-tests.

## Supplementary Material

Reviewer comments

## References

[1] Arvaniti V, D’Amico G, Fede G, Manousou P, Tsochatzis E, Pleguezuelo M, Burroughs AK (2010) Infections in patients with cirrhosis increase mortality four-fold and should be used in determining prognosis. Gastroenterology 139: 1246–1256.e5. 10.1053/j.gastro.2010.06.019 20558165

[2] Tandon P, Garcia-Tsao G (2008) Bacterial infections, sepsis, and multiorgan failure in cirrhosis. Semin Liver Dis 28: 26–42. 10.1055/s-2008-1040319 18293275

[3] Fernández J, Navasa M, Gómez J, Colmenero J, Vila J, Arroyo V, Rodés J (2002) Bacterial infections in cirrhosis: Epidemiological changes with invasive procedures and norfloxacin prophylaxis. Hepatology 35: 140–148. 10.1053/jhep.2002.30082 11786970

[4] Borzio M, Salerno F, Piantoni L, Cazzaniga M, Angeli P, Bissoli F, Boccia S, Colloredo-Mels G, Corigliano P, Fornaciari G, (2001) Bacterial infection in patients with advanced cirrhosis: A multicentre prospective study. Dig Liver Dis 33: 41–48. 10.1016/s1590-8658(01)80134-1 11303974

[5] Moreau R, Jalan R, Gines P, Pavesi M, Angeli P, Cordoba J, Durand F, Gustot T, Saliba F, Domenicali M, (2013) Acute-on-chronic liver failure is a distinct syndrome that develops in patients with acute decompensation of cirrhosis. Gastroenterology 144: 1426–1437.e9. 10.1053/j.gastro.2013.02.042 23474284

[6] Fernández J, Acevedo J, Wiest R, Gustot T, Amoros A, Deulofeu C, Reverter E, Martínez J, Saliba F, Jalan R, (2018) Bacterial and fungal infections in acute-on-chronic liver failure: Prevalence, characteristics and impact on prognosis. Gut 67: 1870–1880. 10.1136/gutjnl-2017-314240 28847867

[7] Bonnel AR, Bunchorntavakul C, Reddy KR (2011) Immune dysfunction and infections in patients with cirrhosis. Clin Gastroenterol Hepatol 9: 727–738. 10.1016/j.cgh.2011.02.031 21397731

[8] Albillos A, Lario M, Álvarez-Mon M (2014) Cirrhosis-associated immune dysfunction: Distinctive features and clinical relevance. J Hepatol 61: 1385–1396. 10.1016/j.jhep.2014.08.010 25135860

[9] Bajaj JS, O’Leary JG, Reddy KR, Wong F, Olson JC, Subramanian RM, Brown G, Noble NA, Thacker LR, Kamath PS, (2012) Second infections independently increase mortality in hospitalized patients with cirrhosis: The North American consortium for the study of end-stage liver disease (NACSELD) experience. Hepatology 56: 2328–2335. 10.1002/hep.25947 22806618 PMC3492528

[10] Wasmuth HE, Kunz D, Yagmur E, Timmer-Stranghöner A, Vidacek D, Siewert E, Bach J, Geier A, Purucker EA, Gressner AM, (2005) Patients with acute on chronic liver failure display “sepsis-like” immune paralysis. J Hepatol 42: 195–201. 10.1016/j.jhep.2004.10.019 15664244

[11] Berres ML, Schnyder B, Yagmur E, Inglis B, Stanzel S, Tischendorf JJW, Koch A, Winograd R, Trautwein C, Wasmuth HE (2009) Longitudinal monocyte human leukocyte antigen-DR expression is a prognostic marker in critically ill patients with decompensated liver cirrhosis. Liver Int 29: 536–543. 10.1111/j.1478-3231.2008.01870.x 18795898

[12] Berry PA, Antoniades CG, Carey I, McPhail MJW, Hussain MJ, Davies ET, Wendon JA, Vergani D (2011) Severity of the compensatory anti-inflammatory response determined by monocyte HLA-DR expression may assist outcome prediction in cirrhosis. Intensive Care Med 37: 453–460. 10.1007/s00134-010-2099-7 21161643

[13] Cirera I, Bauer TM, Navasa M, Vila J, Grande L, Taurá P, Fuster J, García-Valdecasas JC, Lacy A, Suárez MJ, (2001) Bacterial translocation of enteric organisms in patients with cirrhosis. J Hepatol 34: 32–37. 10.1016/s0168-8278(00)00013-1 11211904

[14] Zapater P, Francés R, González-Navajas JM, de la Hoz MA, Moreu R, Pascual S, Monfort D, Montoliu S, Vila C, Escudero A, (2008) Serum and ascitic fluid bacterial DNA: A new independent prognostic factor in noninfected patients with cirrhosis. Hepatology 48: 1924–1931. 10.1002/hep.22564 19003911

[15] Lemke G, Rothlin CV (2008) Immunobiology of the TAM receptors. Nat Rev Immunol 8: 327–336. 10.1038/nri2303 18421305 PMC2856445

[16] Rothlin CV, Ghosh S, Zuniga EI, Oldstone MBA, Lemke G (2007) TAM receptors are pleiotropic inhibitors of the innate immune response. Cell 131: 1124–1136. 10.1016/j.cell.2007.10.034 18083102

[17] Tsou WI, Nguyen KQN, Calarese DA, Garforth SJ, Antes AL, Smirnov SV, Almo SC, Birge RB, Kotenko SV (2014) Receptor tyrosine kinases, TYRO3, AXL, and MER, demonstrate distinct patterns and complex regulation of ligand-induced activation. J Biol Chem 289: 25750–25763. 10.1074/jbc.m114.569020 25074926 PMC4162177

[18] Bernsmeier C, Pop OT, Singanayagam A, Triantafyllou E, Patel VC, Weston CJ, Curbishley S, Sadiq F, Vergis N, Khamri W, (2015) Patients with acute-on-chronic liver failure have increased numbers of regulatory immune cells expressing the receptor tyrosine kinase MERTK. Gastroenterology 148: 603–615.e14. 10.1053/j.gastro.2014.11.045 25479139

[19] Triantafyllou E, Pop OT, Possamai LA, Wilhelm A, Liaskou E, Singanayagam A, Bernsmeier C, Khamri W, Petts G, Dargue R, (2018) MerTK expressing hepatic macrophages promote the resolution of inflammation in acute liver failure. Gut 67: 333–347. 10.1136/gutjnl-2016-313615 28450389 PMC5868289

[20] Bernsmeier C, Triantafyllou E, Brenig R, Lebosse FJ, Singanayagam A, Patel VC, Pop OT, Khamri W, Nathwani R, Tidswell R, (2018) CD14+ CD15− HLA-DR− myeloid-derived suppressor cells impair antimicrobial responses in patients with acute-on-chronic liver failure. Gut 67: 1155–1167. 10.1136/gutjnl-2017-314184 28592438 PMC5969362

[21] D’Amico G, Garcia-Tsao G, Pagliaro L (2006) Natural history and prognostic indicators of survival in cirrhosis: A systematic review of 118 studies. J Hepatol 44: 217–231. 10.1016/j.jhep.2005.10.013 16298014

[22] Bronte V, Brandau S, Chen SH, Colombo MP, Frey AB, Greten TF, Mandruzzato S, Murray PJ, Ochoa A, Ostrand-Rosenberg S, (2016) Recommendations for myeloid-derived suppressor cell nomenclature and characterization standards. Nat Commun 7: 12150 10.1038/ncomms12150 27381735 PMC4935811

[23] Bernardi M, Moreau R, Angeli P, Schnabl B, Arroyo V (2015) Mechanisms of decompensation and organ failure in cirrhosis: From peripheral arterial vasodilation to systemic inflammation hypothesis. J Hepatol 63: 1272–1284. 10.1016/j.jhep.2015.07.004 26192220

[24] Trahtemberg U, Mevorach D (2017) Apoptotic cells induced signaling for immune homeostasis in macrophages and dendritic cells. Front Immunol 8: 1356 10.3389/fimmu.2017.01356 29118755 PMC5661053

[25] Arandjelovic S, Ravichandran KS (2015) Phagocytosis of apoptotic cells in homeostasis. Nat Immunol 16: 907–917. 10.1038/ni.3253 26287597 PMC4826466

[26] Zizzo G, Hilliard BA, Monestier M, Cohen PL (2012) Efficient clearance of early apoptotic cells by human macrophages requires M2c polarization and MerTK induction. J Immunol 189: 3508–3520. 10.4049/jimmunol.1200662 22942426 PMC3465703

[27] Holland SJ, Pan A, Franci C, Hu Y, Chang B, Li W, Duan M, Torneros A, Yu J, Heckrodt TJ, (2010) R428, a selective small molecule inhibitor of Axl kinase, blocks tumor spread and prolongs survival in models of metastatic breast cancer. Cancer Res 70: 1544–1554. 10.1158/0008-5472.can-09-2997 20145120

[28] Kim NY, Lee HY, Lee C (2015) Metformin targets Axl and Tyro3 receptor tyrosine kinases to inhibit cell proliferation and overcome chemoresistance in ovarian cancer cells. Int J Oncol 47: 353–360. 10.3892/ijo.2015.3004 25975389

[29] Bansal N, Petrie K, Christova R, Chung CY, Leibovitch BA, Howell L, Gil V, Sbirkov Y, Lee E, Wexler J, (2015) Targeting the SIN3A-PF1 interaction inhibits epithelial to mesenchymal transition and maintenance of a stem cell phenotype in triple negative breast cancer. Oncotarget 6: 34087–34105. 10.18632/oncotarget.6048 26460951 PMC4741438

[30] Fujimori T, Kato K, Fujihara S, Iwama H, Yamashita T, Kobayashi K, Kamada H, Morishita A, Kobara H, Mori H, (2015) Antitumor effect of metformin on cholangiocarcinoma: In vitro and in vivo studies. Oncol Rep 34: 2987–2996. 10.3892/or.2015.4284 26398221

[31] Müzes G, Deák G, Láng I, González-Cabello R, Gergely P, Fehér J (1989) Depressed monocyte production of interleukin-1 and tumor necrosis factor-alpha in patients with alcoholic liver cirrhosis. Liver 9: 302–306. 10.1111/j.1600-0676.1989.tb00415.x 2586242

[32] von Baehr V, Docke W, Plauth M, Liebenthal C, Kupferling S, Lochs H, Baumgarten R, Volk H (2000) Mechanisms of endotoxin tolerance in patients with alcoholic liver cirrhosis: Role of interleukin 10, interleukin 1 receptor antagonist, and soluble tumour necrosis factor receptors as well as effector cell desensitisation. Gut 47: 281–287. 10.1136/gut.47.2.281 10896923 PMC1728013

[33] Zimmermann HW, Seidler S, Nattermann J, Gassler N, Hellerbrand C, Zernecke A, Tischendorf JJW, Luedde T, Weiskirchen R, Trautwein C, (2010) Functional contribution of elevated circulating and hepatic non-classical CD14CD16 monocytes to inflammation and human liver fibrosis. PLoS One 5: e11049 10.1371/journal.pone.0011049 20548789 PMC2883575

[34] Zagórska A, Través PG, Lew ED, Dransfield I, Lemke G (2014) Diversification of TAM receptor tyrosine kinase function. Nat Immunol 15: 920–928. 10.1038/ni.2986 25194421 PMC4169336

[35] Fujimori T, Grabiec AM, Kaur M, Bell TJ, Fujino N, Cook PC, Svedberg FR, MacDonald AS, Maciewicz RA, Singh D, (2015) The Axl receptor tyrosine kinase is a discriminator of macrophage function in the inflamed lung. Mucosal Immunol 8: 1021–1030. 10.1038/mi.2014.129 25603826 PMC4430298

[36] Hernandez C, Huebener P, Pradere JP, Friedman RA, Schwabe RF (2019) HMGB1 links chronic liver injury to progenitor responses and hepatocarcinogenesis. J Clin Invest 128: 2436–2450. 10.1172/JCI128262 PMC5983315 29558367

[37] Gay CM, Balaji K, Byers LA (2017) Giving AXL the axe: Targeting AXL in human malignancy. Br J Cancer 116: 415–423. 10.1038/bjc.2016.428 28072762 PMC5318970

[38] Bárcena C, Stefanovic M, Tutusaus A, Joannas L, Menéndez A, García-Ruiz C, Sancho-Bru P, Marí M, Caballeria J, Rothlin CV, (2015) Gas6/Axl pathway is activated in chronic liver disease and its targeting reduces fibrosis via hepatic stellate cell inactivation. J Hepatol 63: 670–678. 10.1016/j.jhep.2015.04.013 25908269 PMC4543529

[39] Espindola MS, Habiel DM, Narayanan R, Jones I, Coelho AL, Murray LA, Jiang D, Noble PW, Hogaboam CM (2018) Targeting of TAM receptors ameliorates fibrotic mechanisms in idiopathic pulmonary fibrosis. Am J Respir Crit Care Med 197: 1443–1456. 10.1164/rccm.201707-1519oc 29634284 PMC6005556

[40] Abou-Alfa GK, Meyer T, Cheng AL, El-Khoueiry AB, Rimassa L, Ryoo BY, Cicin I, Merle P, Chen Y, Park JW, (2018) Cabozantinib in patients with advanced and progressing hepatocellular carcinoma. N Engl J Med 379: 54–63. 10.1056/nejmoa1717002 29972759 PMC7523244

[41] Kim J, Kwak HJ, Cha JY, Jeong YS, Rhee SD, Kim KR, Cheon HG (2014) Metformin suppresses lipopolysaccharide (LPS)-induced inflammatory response in murine macrophages via activating transcription factor-3 (ATF-3) induction. J Biol Chem 289: 23246–23255. 10.1074/jbc.m114.577908 24973221 PMC4132821

[42] Ursini F, Russo E, Pellino G, D’Angelo S, Chiaravalloti A, De Sarro G, Manfredini R, De Giorgio R (2018) Metformin and autoimmunity: A “new deal” of an old drug. Front Immunol 9: 1236 10.3389/fimmu.2018.01236 29915588 PMC5994909

[43] Nkontchou G, Cosson E, Aout M, Mahmoudi A, Bourcier V, Charif I, Ganne-Carrie N, Grando-Lemaire V, Vicaut E, Trinchet JC, (2011) Impact of metformin on the prognosis of cirrhosis induced by viral hepatitis C in diabetic patients. J Clin Endocrinol Metab 96: 2601–2608. 10.1210/jc.2010-2415 21752887

[44] Tripathi DM, Erice E, Lafoz E, García-Calderó H, Sarin SK, Bosch J, Gracia-Sancho J, García-Pagán JC (2015) Metformin reduces hepatic resistance and portal pressure in cirrhotic rats. Am J Physiol Gastrointest Liver Physiol 309: G301–G309. 10.1152/ajpgi.00010.2015 26138461

[45] Dengler M, Staufer K, Huber H, Stauber R, Bantel H, Weiss KH, Starlinger P, Pock H, Klöters-Plachky P, Gotthardt DN, (2017) Soluble Axl is an accurate biomarker of cirrhosis and hepatocellular carcinoma development: Results from a large scale multicenter analysis. Oncotarget 8: 46234–46248. 10.18632/oncotarget.17598 28526812 PMC5542263

[46] Staufer K, Dengler M, Huber H, Marculescu R, Stauber R, Lackner C, Dienes H-P, Kivaranovic D, Schachner C, Zeitlinger M, (2017) The non-invasive serum biomarker soluble Axl accurately detects advanced liver fibrosis and cirrhosis. Cell Death Dis 8: e3135 10.1038/cddis.2017.554 29072690 PMC5680921

[47] Jalan R, Pavesi M, Saliba F, Amorós A, Fernandez J, Holland-Fischer P, Sawhney R, Mookerjee R, Caraceni P, Moreau R, (2015) The CLIF consortium acute decompensation score (CLIF-C ADs) for prognosis of hospitalised cirrhotic patients without acute-on-chronic liver failure. J Hepatol 62: 831–840. 10.1016/j.jhep.2014.11.012 25463539

[48] Abeles RD, McPhail MJ, Sowter D, Antoniades CG, Vergis N, Vijay GKM, Xystrakis E, Khamri W, Shawcross DL, Ma Y, (2012) CD14, CD16 and HLA-DR reliably identifies human monocytes and their subsets in the context of pathologically reduced HLA-DR expression by CD14hi/CD16neg monocytes: Expansion of CD14hi/CD16pos and contraction of CD14lo/CD16pos monocytes in acute liver failure. Cytometry A 81: 823–834. 10.1002/cyto.a.22104 22837127

[49] Dehio M, Knorre A, Lanz C, Dehio C (1998) Construction of versatile high-level expression vectors for Bartonella henselae and the use of green fluorescent protein as a new expression marker. Gene 215: 223–229. 10.1016/s0378-1119(98)00319-9 9714815

[50] Van der Maaten L, Hinton G (2008) Visualizing data using t-SNE. J Mach Learn Res 9: 2579–2605.

